# Comparative Evaluation of Novel ^177^Lu-Labeled PNA Probes for Affibody-Mediated PNA-Based Pretargeting

**DOI:** 10.3390/cancers13030500

**Published:** 2021-01-28

**Authors:** Hanna Tano, Maryam Oroujeni, Anzhelika Vorobyeva, Kristina Westerlund, Yongsheng Liu, Tianqi Xu, Daniel Vasconcelos, Anna Orlova, Amelie Eriksson Karlström, Vladimir Tolmachev

**Affiliations:** 1 Department of Protein Science, School of Engineering Sciences in Chemistry, Biotechnology and Health, KTH Royal Institute of Technology, 106 91 Stockholm, Sweden; htano@kth.se (H.T.); krw@kth.se (K.W.); danielv@med.up.pt (D.V.); 2 Department of Immunology, Genetics and Pathology, Dag Hammarskjölds väg 20, Uppsala University, 751 85 Uppsala, Sweden; maryam.oroujeni@igp.uu.se (M.O.); anzhelika.vorobyeva@igp.uu.se (A.V.); yongsheng.liu@igp.uu.se (Y.L.); tianqi.xu@igp.uu.se (T.X.); vladimir.tolmachev@igp.uu.se (V.T.); 3 Research Centrum for Oncotheranostics, Research School of Chemistry and Applied Biomedical Sciences, Tomsk Polytechnic University, 634050 Tomsk, Russia; anna.orlova@ilk.uu.se; 4 Department of Medicinal Chemistry, Dag Hammarskjölds väg 14C, Uppsala University, 751 23 Uppsala, Sweden

**Keywords:** pretargeting, PNA, affibody molecules, radionuclide therapy, HER2-expressing xenografts

## Abstract

**Simple Summary:**

Affibody molecules are small, engineered affinity proteins based on a nonimmunoglobulin scaffold. Affibody-based radionuclide imaging probes have demonstrated excellent tumor targeting. However, the renal clearance of affibody molecules is accompanied by high reabsorption and retention of activity in the kidney, which prevents their use for radionuclide therapy. We have previously shown the feasibility of overcoming the high renal uptake using a pretargeting approach for affibody-mediated therapy based on peptide nucleic acid (PNA) hybridization. In this study, we test the hypothesis that shortening the PNA pretargeting probes would further increase the difference between the accumulation of radiometals in tumor xenografts and in kidneys. A series of novel PNA probes has been designed and evaluated in vitro and in vivo. We have found that a variant containing 9 nucleobases enables a two-fold increase of the tumor-to-kidney dose ratio compared with a variant containing 15 nucleobases. This creates preconditions for more efficient therapy of cancer.

**Abstract:**

Affibody-mediated PNA-based pretargeting is a promising approach to radionuclide therapy of HER2-expressing tumors. In this study, we test the hypothesis that shortening the PNA pretargeting probes would increase the tumor-to-kidney dose ratio. The primary probe Z_HER2:342_-SR-*HP15* and the complementary secondary probes *HP16*, *HP17*, and *HP18*, containing 9, 12, and 15 nucleobases, respectively, and carrying a 1,4,7,10-tetraazacyclododecane-1,4,7,10-tetraacetic acid (DOTA) chelator were designed, synthesized, characterized in vitro, and labeled with ^177^Lu. In vitro pretargeting was studied in HER2-expressing SKOV3 and BT474 cell lines. The biodistribution of these novel probes was evaluated in immunodeficient mice bearing SKOV3 xenografts and compared to the previously studied [^177^Lu]Lu-*HP2*. Characterization confirmed the formation of high-affinity duplexes between *HP15* and the secondary probes, with the affinity correlating with the length of the complementary PNA sequences. All the PNA-based probes were bound specifically to HER2-expressing cells in vitro. In vivo studies demonstrated HER2-specific uptake of all ^177^Lu-labeled probes in xenografts in a pretargeting setting. The ratio of cumulated radioactivity in the tumor to the radioactivity in kidneys was dependent on the secondary probe’s size and decreased with an increased number of nucleobases. The shortest PNA probe, [^177^Lu]Lu-*HP16*, showed the highest tumor-to-kidney ratio. [^177^Lu]Lu-*HP16* is the most promising secondary probe for affibody-mediated tumor pretargeting.

## 1. Introduction

Affibody molecules are small (molecular weight 7 kDa), engineered scaffold proteins that can be selected to bind with high affinity to a broad spectrum of biomolecules [1]; they belong to a class of engineered scaffold proteins with potential for cancer diagnostics and therapy [2]. Affibody molecules are well suited as radionuclide imaging probes due to their small size, high affinity, and selectivity to cancer-associated targets [1]. Another advantage of affibody molecules is the high-yield recombinant production in prokaryotes. Affibody-based imaging probes for epidermal growth factor receptor (EGFR or HER1), human epidermal growth factor receptor type 2 (HER2), human epidermal growth factor receptor type 3 (HER3), platelet-derived growth factor receptor β (PDGFRβ), insulin-like growth factor-1 receptor (IGF-1R), vascular endothelial growth factor receptor 2 (VEGFR2), and programmed death ligand 1 (PD-L1) have demonstrated very promising features in preclinical experiments [3]. Furthermore, excellent imaging of HER2 has been demonstrated in clinics [4,5]. 

The targeting of HER2 using monoclonal antibodies and antibody–drug conjugates extends the survival of breast and gastroesophageal cancer patients. However, an onset of resistance to such therapies is inevitable despite preserved HER2 expression [6,7]. A HER2-targeted radionuclide therapy might be a solution in this case. However, the mainstream approach to targeted radionuclide therapy, i.e., the use of radiolabeled monoclonal antibodies, is inefficient in solid tumors due to their long residence in circulation, causing excessive irradiation of bone marrow [8]. Direct application of affibody molecules for radionuclide therapy is complicated due to high renal reabsorption and long retention of activity in the case of radiometal labels [9]. In this case, the renal uptake exceeded tumor uptake by 10–20 fold [3]. Common methods applied for the reduction of renal uptake of radiolabeled proteins and peptides have turned out to be inefficient for affibody molecules [10,11]. 

Our solution to the problem of high renal reabsorption of radiolabeled affibody molecules is applying pretargeting, a methodology that separates the acts of molecular recognition of cancer-associated abnormalities and radionuclide delivery [12,13]. In pretargeting, a target-specific primary agent, coupled to a recognition tag, is injected to localize in the tumor. After clearance of the primary agent from blood, a radiolabeled secondary probe with a high affinity to the recognition tag is injected. Low uptake of the secondary probe in kidneys is critical for successful affibody-based pretargeted therapy. Affibody molecules are attractive candidates as primary probes because they clear rapidly from blood and are slowly internalized by cancer cells [14]. Importantly, affibody molecules are rapidly internalized by proximal tubule epithelial cells after renal reabsorption [3]. Thus, affibody-based primary probes disappear from the lumen of the proximal tubule and cannot react with secondary probes in urine. After an evaluation of different approaches [15,16], the hybridization of complementary peptide nucleic acid (PNA) probes was selected for affibody-based pretargeting as it provided the best retention of activity in tumors. PNA is a class of synthetic DNA analogs capable of Watson–Crick base-pairing [17,18]. The PNA backbone is built up of repeating N-(2-aminoethyl)-glycine units connected by amide bonds, and purine and pyrimidine nucleobases are connected to this scaffold via carboxymethyl linkers. PNAs are resistant to degradation by nucleases and proteases and have shown excellent stability in human blood serum [19]. They are nonimmunogenic and have low general toxicity. The molecular design of the first-generation of the primary agent Z_HER2:342_-SR-*HP1* and the secondary probe *HP2* was successful as it provided high affinity and specificity of PNA hybridization, specific accumulation of the primary probes in tumors, and efficient specific delivery of radiometals [16,20]. Labeling of *HP2* with ^177^Lu [13,21], ^111^In [16,20], and ^68^Ga [22] resulted in appreciably higher uptake in tumors than in kidneys, although the renal uptake was the highest among normal tissues. Experimental therapy using the Z_HER2:342_-SR-*HP1*/[^177^Lu]Lu-*HP2* pretargeting system significantly increased the median survival of mice bearing HER2-expressing xenografts (66 days for the treated group compared to 32 days for [^177^Lu]Lu-*HP2* only) without observable bone marrow and renal toxicity [21]. However, a further increase in the ratio of absorbed dose to tumor, compared to doses to normal tissues, first and foremost to the kidneys, is necessary to obtain a curative effect of such treatment. 

A possible optimization parameter is the length of the secondary probe. Intuitively, a reduction of the length would reduce the hydrodynamic radius of the probe, which might facilitate both its extravasation and diffusion in the tumor interstitium, improving both the localization in tumors and the uniformity of distribution inside the tumor. There are, however, apparent risks associated with a reduction of the secondary probe’s size. First, a reduction of the number of nucleobases might decrease the strength of hybridization with the primary probe. Second, modification of the base composition might affect off-target interactions, resulting in elevated uptake in normal tissues. For example, minor structural changes associated with the substitution of ^177^Lu by ^111^In or ^68^Ga resulted in significant differences in renal uptake [22] or uptake in blood, liver, and bone [23]. The biodistribution is further dependent not only on the number and nature of nucleobases but also on their order in a PNA sequence. The scrambling of nucleobases in ^99m^Tc-labeled antisense PNA binding to mRNA encoding MYC protein resulted in more than a two-fold decrease of uptake in normal tissues [24,25]. Thus, experimental in vivo studies were required to evaluate if the second-generation secondary probes would provide a better biodistribution and dosimetry profile. 

**Figure 1:**
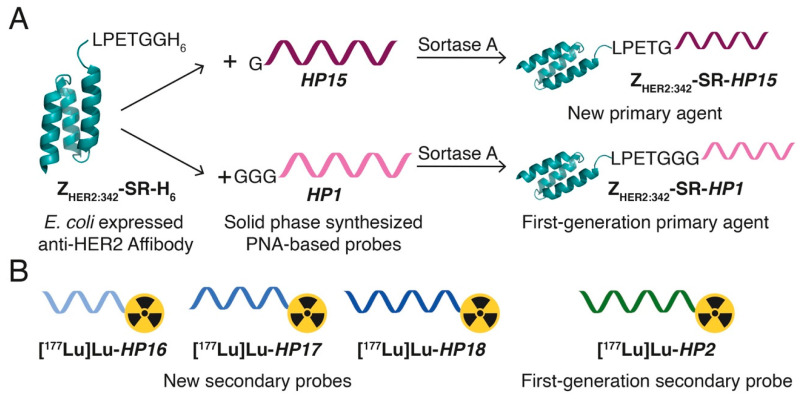
Schematic illustration of the (**A**) primary agents and (**B**) secondary probes used in this study (for PNA probe sequences, see Table 1). (**A**) The new primary agent, Z_HER2:342-_SR-*HP15*, was produced using sortase-catalyzed ligation of a solid phase-synthesized PNA-based probe, *HP15*, to an *E. coli*-expressed anti-HER2 affibody molecule, Z_HER2:342_-SR-H_6_. The resulting chimeric Z_HER2:342-_SR-*HP15* was designed to have the ability to simultaneously bind to HER2-overexpressing tumor cells and to one of the new complementary secondary probes, *HP16*, *HP17*, or *HP18*, with high affinity. For comparative biodistribution studies, the first-generation primary agent Z_HER2:342-_SR-*HP1* was produced using the same protocol for Z_HER2:342-_SR-*HP15*. Z_HER2:342-_SR-*HP15* and Z_HER2:342-_SR-*HP1* contain the same affibody molecule and differ only in the sequence of the attached PNA-based probe. (**B**) The three new secondary probes, *HP16* (9-mer PNA), *HP17* (12-mer PNA), and *HP18* (15-mer PNA), were designed to bind to Z_HER2:342-_SR-*HP15* (15-mer PNA) through sequence-specific PNA:PNA hybridization. The new secondary probes were synthesized using solid phase synthesis, and all carry a DOTA chelator for radiometal labeling with, in this study, ^177^Lu. For biodistribution studies in vivo, the first-generation secondary probe *HP2* was produced and ^177^Lu-labeled using the same protocols for the new secondary probes.

**Figure 2:**
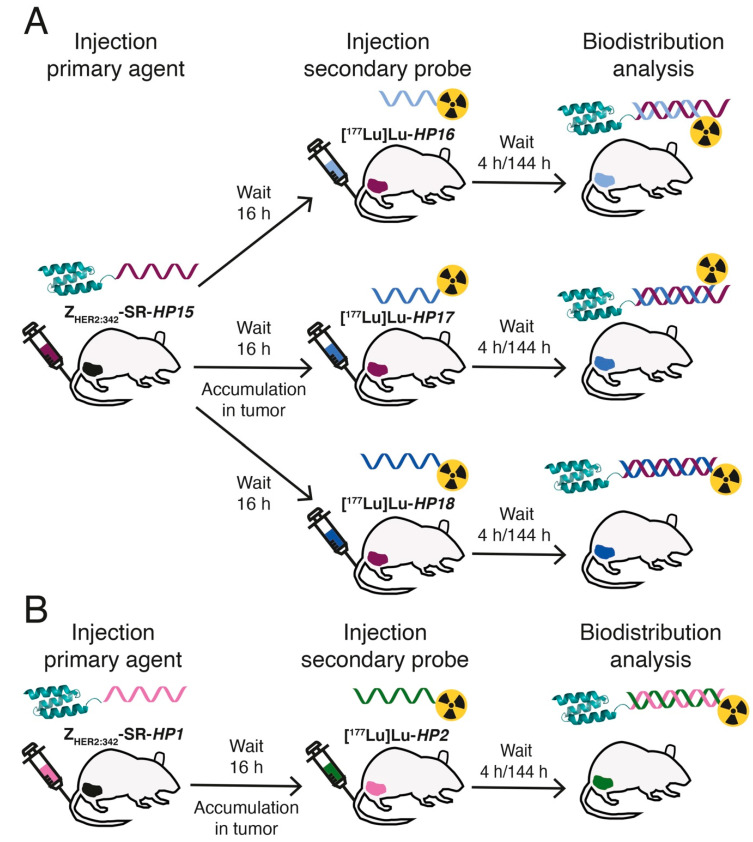
Schematic representation of the pretargeting protocol used for in vivo biodistribution analysis of the new ^177^Lu-labeled secondary probes. (**A**) Thirty mice carrying HER2-overexpressing SKOV3 xenografts in their right hind legs received an injection of Z_HER2:342_-SR-*HP15* in the tail vein. The mice were randomly split into three groups of ten mice, and, after a 16 h waiting period, all mice received a second injection of a ^177^Lu-labeled secondary probe—[^177^Lu]Lu-*HP16*, [^177^Lu]Lu-*HP17*, or [^177^Lu]Lu-*HP18*. All mice were euthanized for biodistribution analysis at 4 h/144 h after injection of the secondary probe. The uptake of radioactivity, expressed as a percentage of the total injected dose per gram of sample (% ID/g) in the organs of interest and the tumor, was then calculated as the average value from five mice. (**B**) As a biodistribution comparison, the uptake of the previously studied first-generation secondary probe, [^177^Lu]Lu-*HP2*, was measured using the same protocol for the new secondary probes described above but with Z_HER2:342_-SR-*HP1* as the primary agent in the first injection.

In this study, we have designed second-generation hybridization probes: the primary agent *HP15* and a set of secondary probes—*HP16* (9-mer PNA), *HP17* (12-mer PNA), and *HP18* (15-mer PNA) (Table 1, Figure 1). The versatile chelator DOTA was incorporated into the secondary probes to enable labeling with a variety of therapeutic nuclides (e.g., ^90^Y, ^177^Lu, ^225^Ac). The same chelator was also incorporated in *HP15* to permit site-specific radiometal labeling of the primary targeting agent, facilitating preclinical cellular processing and biodistribution studies and clinical pharmacokinetics evaluation. The specificity and strength of pretargeting, as well as the cellular processing of the primary agent and secondary probes, were evaluated in vitro. Biodistribution of the new ^177^Lu-labeled secondary probes and pretargeting specificity were evaluated in vivo and compared with the biodistribution of the first-generation secondary probe [^177^Lu]Lu-*HP2* (Figure 2). For estimation of absorbed doses to tumors and kidneys, a clinically validated two-time point approach was used [26].

## 2. Results

### 2.1. Production and Characterization of the Affibody–PNA Conjugate and the Complementary PNA Probes

Affibody Z_HER2:342_-SR-H_6_ and *HP15* were conjugated using Sortase A^3*^. The ligation efficiency for the reaction was estimated to 40% based on integrated areas under peaks at 260 nm in RP-HPLC (Figure S7). A ligation efficiency of 40% is lower than the previously reported ligation efficiency of 70% [13] for conjugation of *HP1* to Z_HER2:342_-SR-H_6_. However, *HP15* has a single glycine residue at the N-terminus, compared to three glycine residues at the N-terminus of *HP1*, which might influence ligation efficiency. The ligation efficiency of 40% is in the same range as the conjugation of *HP1* using wild-type sortase A, which was reported to be 45% [20]. 

**Figure 3:**
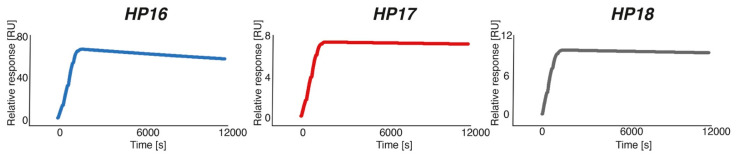
Surface plasmon resonance (SPR) sensorgrams of single-cycle kinetic titration of *HP16*, *HP17*, and *HP18* binding to immobilized Z_HER2:342_-SR-*HP15*. Each PNA probe was injected at concentrations of 22.6, 45.3, 90.6, 181.25, and 362.5 nM.

Hybridization of the three complementary PNA probes to immobilized Z_HER2:342_-SR-*HP15* was analyzed by surface plasmon resonance (SPR). Representative sensorgrams of the interactions, analyzed using single-cycle injection, are shown in Figure 3. The association rate constants, k_a_, were estimated to be 4.6 × 10^4^, 4.3 × 10^4^, and 5.7 × 10^4^ M^−1^s^−1^ for *HP16*, *HP17*, and *HP18*, respectively. Hence, the on-rates for all three PNA probes, interacting with Z_HER2:342_-SR-*HP15*, are within the same range. The dissociation rate constant, k_d_, was estimated to 1.2 × 10^−5^ s^−1^ for *HP16*, whereas for the other two complementary PNA probes, the k_d_ was too slow to be determined using Biacore 8K. The equilibrium dissociation constant for *HP16* was calculated to be approximately 280 pM (Table 2), while the affinity for *HP17* and *HP18* to Z_HER2:342_-SR-*HP15* was estimated to be higher. 

**Figure 4:**
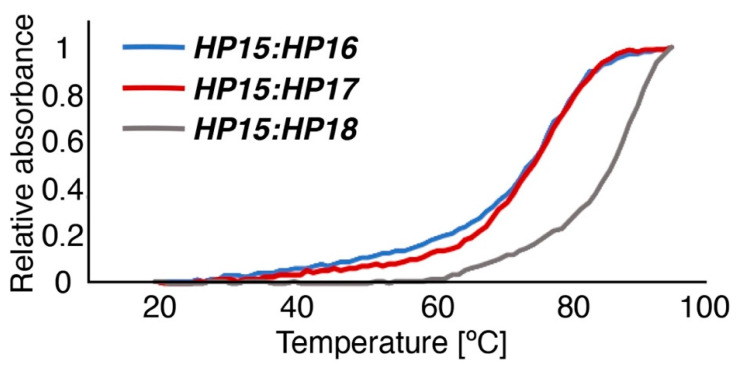
Normalized melting temperature curves shown for the three PNA-hybridization complexes *HP15*:*HP16* (blue), *HP15*:*HP17* (red), and *HP15*:*HP18* (grey).

Melting temperatures for each PNA hybridization probe, after duplex formation with *HP15*, were monitored at 260 nm and estimated to 73, 75, and 87 °C for *HP16*, *HP17*, and *HP18*, respectively (Figure 4). The spectroscopic characterization illustrates that *HP16*, *HP17*, and *HP18* all form duplexes of high thermal stability with the primary PNA probe *HP15*. The lower melting temperatures for *HP16* and *HP17* are expected because of their shorter oligomer lengths compared to *HP18*. 

### 2.2. Radiolabeling and In Vitro Stability

Table 3 shows the results of radiolabeling of all probes with ^177^Lu. The radiochemical yield for the new secondary agents after EDTA treatment was over 98%. Therefore, no further purification using NAP-5 was performed for in vitro and in vivo studies. Purification of [^177^Lu]Lu-Z_HER2:342_-SR-*HP15* and [^177^Lu]Lu-*HP2* using a NAP-5 column resulted in 100 ± 0% radiochemical purity for both labels. All probes labeled with ^177^Lu were stable in PBS and in the presence of EDTA for up to 1 h incubation (Table 3).

To validate the radio-ITLC results (Figure S11), radio-HPLC identity was performed, and it demonstrated that no fragmentation occurred after labeling and purification (Figure S12). The radio-HPLC retention time of all probes was around 5.8 min. The retention time of the nonlabeled probes was the same as the labeled ones.

### 2.3. In Vitro Studies

**Figure 5:**
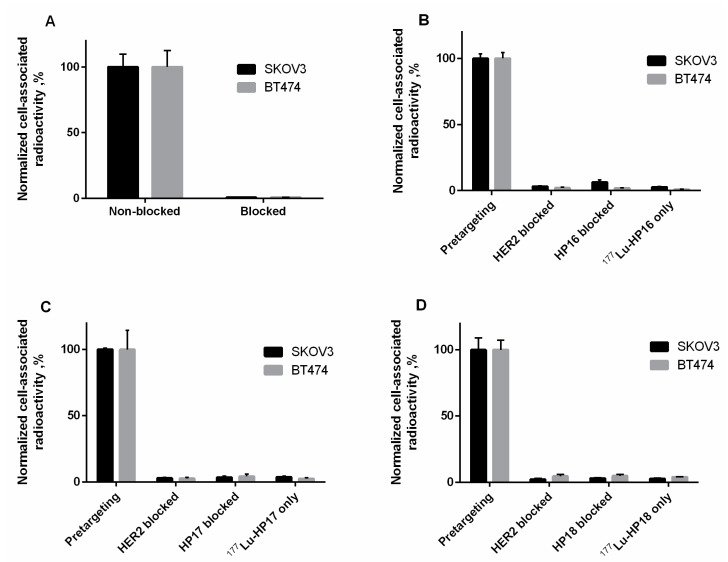
(**A**) In vitro binding specificity of the primary agent [^177^Lu]Lu-Z_HER2:342_-SR-*HP15*, (**B**) [^177^Lu]Lu-*HP16*, (**C**) [^177^Lu]Lu-*HP17*, and (**D**) [^177^Lu]Lu-*HP18* on SKOV3 and BT474. The data are presented as an average value from 3 samples ± SD.

HER2-binding specificity of the primary agent [^177^Lu]Lu-Z_HER2:342_-SR-*HP15* was tested using a saturation experiment. The binding was significantly (*p* < 0.0005) decreased when the cells were presaturated with the anti-HER2 affibody molecule (Figure 5A), demonstrating that the binding was HER2-mediated. 

The specificity of [^177^Lu]Lu-*HP16*, [^177^Lu]Lu-*HP17*, and [^177^Lu]Lu-*HP18* in vitro binding to Z_HER2:342_-SR-*HP15*-pretreated HER2-expressing cells was evaluated (Figure 5B–D). The binding of all secondary agents was significantly (*p* < 0.0005) decreased when HER2 receptors were saturated with the anti-HER2 affibody molecule or when Z_HER2:342_-SR-*HP15*-treated cells were preincubated with a large excess of nonlabeled secondary agents. The binding of all secondary agents to cells without preincubation with the primary agent was significantly (*p* < 0.0005) lower compared to pretargeting. The specificity of [^177^Lu]Lu-*HP2* has been demonstrated earlier [13].

The data concerning the binding affinity of all radiolabeled secondary probes to SKOV3 cells are presented in Table S1 and Figure S13. According to LigandTracer measurements, the best fit of the binding of all the radiolabeled variants to the SKOV3 cell line was achieved using a 1:1 model. Interaction Map calculation showed a rapid association followed by very slow dissociation for [^177^Lu]Lu-Z_HER2:342_-SR-*HP15* and pretargeted ^177^Lu-secondary probes, resulting in picomolar dissociation constants at equilibrium (K_D_). K_D_ values were between 11 and 12 pM. 

Cellular processing and retention of all radiolabels by SKOV3 and BT474 cells after interrupted incubation are shown in Figure S14. For [^177^Lu]Lu-Z_HER2:342_-SR-*HP15* (Figure S14A,B), the internalization was slow, which is typical for HER2-binding affibody molecules and their derivatives. The internalized fraction of [^177^Lu]Lu-Z_HER2:342_-SR-*HP15* was slightly higher in SKOV3 cells than BT474 cells (18 ± 2% for SKOV3 vs. 12 ± 2% for BT474 at 24 h time point). The pattern of internalization of labeled secondary probes after their binding to cells pretreated with a primary probe was similar to the pattern of [^177^Lu]Lu-Z_HER2:342_-SR-*HP15*. Retention of radioactivity over time was highest for [^177^Lu]Lu-Z_HER2:342_-SR-*HP15*, while ^177^Lu-labeled secondary probes showed faster release of bound radioactivity over time. 

### 2.4. In Vivo Studies

The data concerning biodistribution of ^177^Lu-labeled secondary probes without the preinjection of a primary probe are provided in Table 4. The biodistribution of [^177^Lu]Lu-*HP16* and [^177^Lu]Lu-*HP17* was very similar, except for a small but significant difference in bone uptake. In comparison with [^177^Lu]Lu-*HP18*, smaller variants had significantly lower uptake in blood, liver, and kidney. [^177^Lu]Lu-*HP17* also had significantly lower uptake in lung and bone. 

**Figure 6:**
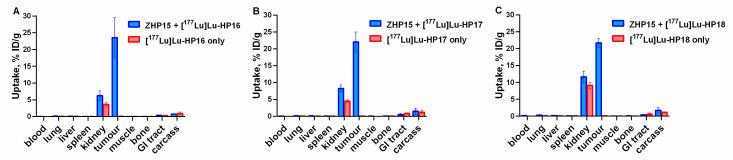
Biodistribution of (**A**) [^177^Lu]Lu-*HP16*, (**B**) [^177^Lu]Lu-*HP17*, and (**C**) [^177^Lu]Lu-*HP18* at 4 h after injection in female Balb/c nu/nu mice bearing SKOV3 xenografts, with and without preinjection of Z_HER2:342_-SR-*HP15*. The uptake is expressed as % ID/g and presented as an average ± SD (*n* = 5).

The results of the in vivo specificity of ^177^Lu-labeled secondary probes (4 h after injection), with and without the preinjection of Z_HER2:342_-SR-*HP15* (4 nmol), are presented in Figure 6. When mice were preinjected with Z_HER2:342_-SR-*HP15*, the tumor uptake of all ^177^Lu-labeled secondary probes was significantly (*p* < 0.00005) higher than without preinjection. Interestingly, uptake in normal tissues was also significantly higher in the case of preinjection of primary probes; however, uptake in normal tissue after preinjection still remained very low.

The results from the comparative biodistribution of [^177^Lu]Lu-*HP16*, [^177^Lu]Lu-*HP17*, and [^177^Lu]Lu-*HP18*, with [^177^Lu]Lu-*HP2* after injections of primary probes in SKOV3-bearing mice, are presented in Table 5. See Figure 2 for a schematic illustration of the pretargeting protocol.

The biodistribution measurements show a rapid clearance from blood and normal organs and tissues for all studied PNA-based probes. Some differences in biodistribution between conjugates were observed. The blood concentration was significantly (*p* < 0.0001) higher for [^177^Lu]Lu-*HP18* than for the other secondary probes at 4 h after injection. At this time point, the hepatic uptakes for [^177^Lu]Lu-*HP16*, [^177^Lu]Lu-*HP17*, and [^177^Lu]Lu-*HP2* (0.1 ± 0.0% ID/g) were equal but significantly (*p* < 0.05) lower than for [^177^Lu]Lu-*HP18* (0.2 ± 0.1% ID/g). The only tissues with prominent uptake were kidney and tumor. The uptake in kidney for [^177^Lu]Lu-*HP16* (6 ± 1% ID/g) was significantly (*p* < 0.05) lower than for [^177^Lu]Lu-*HP18* (12 ± 2% ID/g) and [^177^Lu]Lu-*HP2* (10 ± 2% ID/g). The tumor uptake values were in the range of 19–24% ID/g, without a significant difference between secondary probes. The combination of low kidney uptake and high uptake in tumor resulted in a higher tumor-to-kidney ratio for [^177^Lu]Lu-*HP16* (tumor uptake was 4-fold higher than renal uptake) at 4 h after injection (Table 6). Renal uptake for [^177^Lu]Lu-*HP16* and [^177^Lu]Lu-*HP17* was significantly (*p* < 0.005) lower than for [^177^Lu]Lu-*HP18*. The order for tumor uptake was [^177^Lu]Lu-*HP17* and [^177^Lu]Lu-*HP18* (both 5 ± 1% ID/g) > [^177^Lu]Lu-*HP2* (4 ± 1% ID/g) > [^177^Lu]Lu-*HP16* (both 3 ± 1% ID/g) at 144 h after injection. [^177^Lu]Lu-*HP17* showed better retention of radioactivity in tumor and faster clearance in kidney (tumor uptake was 7-fold higher than renal uptake), resulting in a higher tumor-to-kidney ratio at 144 h after injection. 

### 2.5. SPECT/CT Imaging

**Figure 7:**
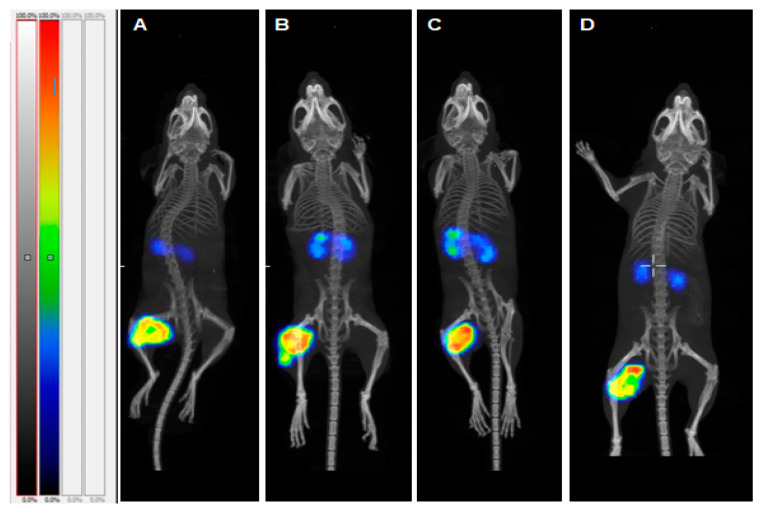
SPECT/CT imaging (maximum intensity projection) of HER2-expressing SKOV3 xenografts for pretargeting using (**A**) [^177^Lu]Lu-*HP16*, (**B**) [^177^Lu]Lu-*HP17*, (**C**) [^177^Lu]Lu-*HP18*, and (**D**) [^177^Lu]Lu-*HP2* 4 h after injection. The color scale bar represents a relative activity normalized to the activity in tissues with the highest uptake in each image.

Results of single photon emission computed tomography/computed tomography (SPECT/CT) imaging (Figure 7) confirmed efficient affibody molecule-based PNA-mediated pretargeting for all variants. The tumors were the sites with the highest uptake. The only tissues with noticeable uptake were kidneys and tumor. The radioactivity uptake in tumor was considerably higher than in kidneys in each animal.

Distribution of radioactivity in tumor was evaluated using autoradiography (Figures S15 and S16). The distribution of activity 4 h after injection was quite uniform and reflected the clustered character of xenografts. At a later time point, a gradient of radioactivity concentration from tumor core to rims was more pronounced. 

The results of the estimated dosimetry to kidney and tumor are shown in Table S2 and Figure S17. The ratio of areas under time-activity plots for tumor and kidneys was 3.8, 2.9, 1.9, and 2.0 for [^177^Lu]Lu-*HP16*, [^177^Lu]Lu-*HP17*, [^177^Lu]Lu-*HP18*, and [^177^Lu]Lu-*HP2*, respectively. This indicates that [^177^Lu]Lu-*HP16* would provide the most favorable dosimetry for therapy application.

## 3. Discussion

Our previously published PNA pretargeting studies were based on PNA probes having 15 complementary bases, resulting in a very strong affinity between the hybridized probes. Measurement of the binding interaction between these probes by SPR-based biosensor analysis showed that the dissociation rate was extremely slow and that less than 5% of hybridized PNA were dissociated during a 17-h long experiment [20]. It is likely that such a high affinity is not necessary and that shorter complementary probes would have a sufficiently high binding affinity for successful in vivo pretargeting. In this study, we evaluated how the length of the PNA sequences in the secondary probes would influence pretargeting efficacy. Instead of simply shortening the previously described secondary probe *HP2*, we decided to redesign a new set of primary and secondary PNA probes and studied their efficacy in affibody-mediated pretargeting. 

Three secondary PNA probes with varying lengths (9-mer, 12-mer and 15-mer) were designed, aiming for an improved biodistribution profile, facilitated production, and improved aqueous solubility while retaining high specificity and affinity between the complementary PNA hybridization probes. The probes were designed to avoid self-complementary sequences as well as extended stretches of purines (A and G), which are known to promote aggregation and be difficult to synthesize and purify after synthesis [27].

Characterization by SPR showed that the new secondary probes associated rapidly with Z_HER2:342_-SR-*HP15* (k_a_ was in the range of 4.3–5.7 × 10^4^ M^−1^s^−1^), although the association rate was slightly lower compared to the rate for Z_HER2:342_-SR-*HP1*-*HP2* hybridization (k_a_ = 1.7 × 10^5^ M^−1^ s^−1^). The *HP17* (12-mer) and *HP18* (15-mer) probes both had such slow dissociation rates after binding to Z_HER2:342_-SR-*HP15* that the rate constants could not reliably be estimated from the SPR analysis. A K_D_ of 280 pM could be determined for the (weaker) interaction between Z_HER2:342_-SR-*HP15* and *HP16* (9-mer), which was expected to be of sufficiently high affinity for the intended pretargeting application. Bispecific monoclonal antibodies binding to the haptens used in pretargeting have been reported to be in the nanomolar affinity range [28]. One example is the earlier demonstrated pretargeting using a bispecific antibody construct binding the tumor-associated antigen CEA and a radiolabeled hapten, with an estimated K_D_ of only 10^−9^–10^−10^ M for binding to the ^111^In-labeled benzyl EDTA derivative [29]. The melting temperatures for duplexes of primary and secondary probes, measured by UV spectroscopy, were nearly equal for *HP15*:*HP18* (87 °C) and *HP1*:*HP2* (86 °C), i.e., the hybridization strength was approximately the same for the 15-mer probes of first and second generations. Compared to *HP15*:*HP18*, the melting temperatures for *HP15*:*HP16* and *HP15*:*HP17* showed a drop in Tm to 73 and 75 °C, respectively, confirming that the stability of the duplexes correlates with the length of the PNA sequences. 

The radiolabeling of the new secondary probes was efficient and stable (Table 3). The binding of [^177^Lu]Lu-Z_HER2:342_-SR-*HP15* to HER2-expressing cell lines in vitro was HER2-specific (Figure 5A). The slow internalization of [^177^Lu]Lu-Z_HER2:342_-SR-*HP15* was favorable for pretargeting application as this enables the long persistence of the primary hybridization probe on the targeted cells’ surface (Figure S14). A set of in vitro tests (Figure 5B–D) demonstrated that pretargeting of new secondary probes in vitro was dependent on the specific binding of primary probes to cells (dramatic reduction of binding in the case of blocking HER2 or in the absence of primary probes) and that interaction between primary and secondary probes was PNA-mediated (dramatic reduction in the case of presaturation with unlabeled PNA). LigandTracer measurements of the kinetics of binding to living SKOV3 cells, which were pretreated with Z_HER2:342_-SR-*HP15*, demonstrated that the binding of all secondary probes was extremely strong. There was no difference in apparent dissociation constants between secondary probes (Table S1). Thus, reduction of length of PNA from 15 to 9 nitrogenous bases was not associated with any observable reduction of their binding to primary probes in the cell assay.

The biodistribution of the new secondary probes without the preinjection of primary probes demonstrated rapid clearance from normal tissues (Table 4). The shorter probes, [^177^Lu]Lu-*HP16* and [^177^Lu]Lu-*HP17*, had lower uptake in blood, liver, and kidneys than [^177^Lu]Lu-*HP18*. A comparison with previously reported radiolabeled PNA–peptide chimeras indicates that our design of secondary probes was quite fortunate. For example, ^111^In-labeled DOTA-anti-bcl-2-PNA-Ala [3,4,5,6], a short peptide-18-mer-PNA chimera [30], had, at 4 h after injection, at least one order of magnitude higher blood uptake and two- to three-fold higher liver uptake compared to our conjugates. The difference was even more striking when compared with ^64^Cu-labeled chimeras [^64^Cu]Cu-DOTA-Y-PNA50-K4 (18-mer) and [^64^Cu]Cu-DOTA-Y-PNA50S-K4 (15-mer), having renal uptake at 4 h after injection of 36.1 ± 3.6% and 60.5 ± 3.6% ID/kidney, respectively [31]. It can be noted that [^64^Cu]Cu-DOTA-Y-PNA50S-K4 had the same length of PNA as [^177^Lu]Lu-*HP18* but a much higher renal uptake. This suggests that the length of PNA is not the only factor determining uptake in normal tissue; the composition of nucleobases and flanking amino acids also play an important role, which has not been sufficiently investigated and will require regular structure–property relationship studies in the future. 

The dramatic increase of uptake of the secondary probes in tumors (Figure 6) after preinjection of primary agents convincingly shows the specificity of pretargeting. Interestingly, an increased uptake of secondary probes after injection of Z_HER2:342_-SR-*HP15* was also observed in blood, kidneys, spleen, and muscles. This might be explained by the association of the secondary probes with the primary agent, which had not completely cleared from circulation or had re-entered the blood flow after dissociation from receptors in the tumor. This effect was less pronounced for [^177^Lu]Lu-*HP16*, which was unexpected as it can be anticipated that the level of the primary agent is the same in all preinjected mice. 

Another interesting observation was the relatively uniform distribution of activity in xenografts for all variants after pretargeting (Figures S15 and S16). The observed heterogeneity was rather associated with the distribution of malignant and connective tissues within a tumor than with the length of PNA in the secondary targeting probe. Thus, macroscopic uniformity of distribution of our [^177^Lu]-labeled probes is not dependent on the length of PNA. It has to be noted that the existing nonuniformity would be compensated by the cross-fire effect.

Tumor uptake exceeded, by several hundred-fold, the uptake in the majority of tissues. It is apparent that only the kidneys would be a dose-limiting organ in a therapeutic context. Thus, the best choice would be a variant that provides the highest ratio between absorbed doses to tumor and to kidneys. A simplified assessment of this was performed by comparing areas under time-activity plots. This estimation suggests that the original [^177^Lu]Lu-*HP2* and the newly designed [^177^Lu]Lu-*HP18* 15-mer-based probes provide similar ratios of doses to tumor and kidneys. The tumor-to-kidney dose ratio for [^177^Lu]Lu-*HP17* (12-mer) was 1.5-fold higher than for [^177^Lu]Lu-*HP2* and [^177^Lu]Lu-*HP18*. [^177^Lu]Lu-*HP16* provided the highest tumor-to-kidney dose ratio. Thus, in the case of a successful overall design of the secondary PNA-based probe, a shorter PNA sequence provides the best dose ratio between tumor and kidneys. Currently, the renal dose limit for ^177^Lu-based radionuclide therapy is considered 28–40 Gy, depending on risks associated with the status of the patient’s renal function [32]. If the preclinical data was translated to humans, the use of [^177^Lu]Lu-*HP16* would result in a tumor-absorbed dose of 120-160 Gy. Such level of tumor dose is associated with a pronounced tumor response during ^177^Lu-based radionuclide therapy in the clinic [33]. An important aspect of the use of our system for radionuclide therapy is a washout of activity from tumors. The tumor-associated activity of [^177^Lu]Lu-*HP16* was reduced from 24 ± 6% ID/g at 4 h to 3 ± 1% ID/g at 144 h after injection. Such washout effect can be observed for other pretargeting systems [34,35] and also for short peptides having short residence in circulation (see, e.g., [36] for somatostatin and [37] for bombesin analogs). This might be explained partially by dissociation of the targeting agent from the cell surface target (in the case of a pretargeting system, this might be the dissociation of the secondary probe from the primary probe or the dissociation of the whole primary–secondary probe complex from the target). The release of internalizing [^177^Lu]-DOTA-TATE [34] from the tumor suggests that a release of intracellular metabolites might play a certain role even in the case of residualizing radiometal labels. In principle, this might be a challenge for the delivery of a sufficient absorbed dose to a tumor. However, the use of Z_HER2:342_-SR-*HP1* in combination with [^177^Lu]Lu-*HP2* provided a significant extension of survival of treated tumor-bearing mice [21]. The profile of activity retention in tumor for that system (17 ± 3% ID/g at 4 h and 3.4 ± 0.6% ID/g at 144 h after injection) was similar to the profile for the Z_HER2:342_-SR-*HP15*:[^177^Lu]Lu-*HP16* system. This suggests that radionuclide therapy using the Z_HER2:342_-SR-*HP15*:[^177^Lu]Lu-*HP16* system is feasible. One or a few additional pretargeting cycles might be necessary to obtain the full effect. Apparently, these additional cycles will not cause an unacceptably high dose to kidneys. 

## 4. Materials and Methods 

### 4.1. Synthesis and Purification of PNA Pretargeting Probes 

Peptide nucleic acid monomers, Fmoc-PNA-A(Bhoc)-OH, Fmoc-PNA-G(Bhoc)-OH, Fmoc-PNA-C(Bhoc)-OH and Fmoc-PNA-T-OH, were purchased from PolyOrg Inc. (Leominster, MA, USA). Rink Amide resin (ChemMatrix, 0.50 mmol/g) was purchased from Biotage (Uppsala, Sweden). 1,4,7,10-Tetraazacyclododecane-1,4,7,10-tetraacetic acid (DOTA) was purchased from CheMatech (Dijon, France). Fmoc-NH-(PEG)_2_-CH_2_COOH (AEEA) was purchased from Merck KGaA (Darmstadt, Germany). Solvents and reagents for solid phase synthesis were obtained from commercial suppliers and used without further purification.

*HP15* was synthesized on a Biotage Initiator+ Alstra microwave peptide synthesizer using Rink Amide resin (ChemMatrix, 0.50 mmol/g) on a 50-μmol scale in a 10-mL reactor vial. Fmoc deprotection was performed at RT in two stages by treating the resin with piperidine–DMF (1:4) for 3 min, followed by piperidine–DMF (1:4) for 10 min. Couplings were performed using 5 eq of PNA or amino acid monomer, 5 eq Oxyma, and 5 eq DIC in DMF. A coupling time of 10 min at 75 °C was used throughout the sequence, followed by a capping step using NMP–lutidine–acetic anhydride (89:6:5) for 2 min.

Orthogonally protected Lys(Mtt) was introduced for the possibility of site-specific introduction of DOTA. Automated synthesis was interrupted after four coupling steps (Fmoc-E-K(Mtt)-[AEEA]-E-resin) for selective side-chain deprotection of Lys(Mtt) with 5–10 additions of fresh TFA:TIS:DCM (1:2:97) followed by 1 min vortexing. Coupling of DOTA was performed using 5 eq of DOTA, 5 eq Oxyma, and 5 eq DIC in DMF at RT for 1 h. After resuming the automated synthesis and completion of all cycles, the resin was washed with DMF, DCM, and, finally, with MeOH and then dried overnight. The PNA–peptide hybrid was cleaved from the solid support using a mixture of TFA:H_2_O:TIS (95:2.5:2.5) for 4 h at RT. The PNA product was finally extracted between diethyl ether and water and lyophilized from the aqueous phase.

The synthesis of the complementary PNA probes *HP16*, *HP17*, and *HP18* was performed as described previously [20]. In brief, coupling was performed with an excess of PNA monomers, benzotriazol-1-yl-oxytripyrrolidinophosphonium hexafluorophosphate (PyBOP; Sigma Aldrich), and the presence of DIEA in NMP and DMF. After coupling, a capping step, followed by the removal of Fmoc-protecting groups, was conducted. After completed synthesis, the PNA probes were cleaved from the resin and ether extracted analogously to *HP15*.

RP-HPLC purification was performed using a semipreparative Zorbax 300 SB-C18 column (9.4 × 250 mm, 5 µm particle size; Agilent, Santa Clara, CA, USA) with a linear gradient of 5–50% B, where A = 0.1% TFA-H_2_O and B = 0.1% TFA-CH_3_CN, for over 25 min, using a flow rate of 3 mL/min, a column temperature of 70 °C, and UV detection at 220 and 260 nm. Collected fractions were analyzed by MALDI-TOF (4800 MALDI-TOF/TOF, AB SCIEX) using an α-cyano-4-hydroxycinnamic acid matrix. Fractions containing the correct products were pooled and lyophilized. 

The purity of *HP16*, *HP17*, and *HP18* was confirmed using analytical RP-HPLC on a Zorbax 300SB-C18 column (4.6 × 150 mm, 3.5 µm particle size; Agilent; Figure S1), followed by MALDI-TOF analysis (Figures S2–S4). Extinction coefficients at 260 nm (ε_260_) were estimated for each PNA probe based on the PNA composition and the extinction coefficient of each PNA monomer (A: 13 700 M^−1^cm^−1^, C: 6 600 M^−1^cm^−1^, G: 11 700 M^−1^cm^−1^, and T: 8 600 M^−1^cm^−1^). Extinction coefficients used throughout all experiments for each probe are the following; *HP16*: 98 000 M^−1^cm^−1^, *HP17*: 126 900 M^−1^cm^−1^, *HP18*: 155 800 M^−1^cm^−1^, and *HP15*: 150 700 M^−1^cm^−1^.

### 4.2. Production of Affibody–PNA Conjugate 

Expression and purification of Z_HER2:342_-SR-H_6_ were based on a previously described method [20]. Briefly, Z_HER2:342_-SR-H_6_ was expressed in *E. coli* BL21(DE3) cells and harvested after induction by IPTG and incubation overnight at RT. Harvested cells were resuspended in a Tris-based binding buffer and subsequently lysed by sonication. Purification was performed using an IMAC matrix (HisPur Cobalt Resin, Thermo Scientific, Rockford, IL, USA), and Z_HER2:342_-SR-H_6_ was eluted in 20 mM Tris-HCl, 300 mM NaCl, and 300 mM imidazole, pH 7.5. Eluted Z_HER2:342_-SR-H_6_ was buffer-exchanged to sortase A conjugation buffer (50 mM Tris-HCl, 150 mM NaCl, 10 mM CaCl_2_, pH 7.5) on a PD-10 desalting column (GE Healthcare, Uppsala, Sweden). The purity and molecular weight of Z_HER2:342_-SR-H_6_ was confirmed using SDS-PAGE and MALDI-TOF (Figures S5 and S6).

Z_HER2:342_-SR-H_6_ was site-specifically conjugated to *HP15* using sortase A-mediated ligation (SML). The SML method described below is based on a protocol for affibody–PNA conjugation previously published by our group [13]. The glycine-modified *HP15* probe was dissolved in 10% DMSO and heated at 80 °C for 5 min before the concentration was estimated. *HP15*, Z_HER2:342_-SR-H_6_, and NiCl_2_ were mixed in sortase A conjugation buffer. The reaction proceeded for 30 min after the addition of Sortase A3*, and the mixture was subsequently subjected to a reverse IMAC step. The conjugation product, hydrolyzed protein byproducts, and unconjugated *HP15* could be collected in the flow-through. Flow-through was buffer-exchanged to 10 mM NaOAc pH 3.6 and lyophilized. The Z_HER2:342_-SR-*HP15* conjugate was purified on RP-HPLC using the same column and solvents for purification of PNA probes but with a gradient going from 5% to 50% B in 25 min; the absorbance was monitored at 220 and 260 nm (Figure S7). Fractions of the Z_HER2:342_-SR-*HP15* conjugate were collected, lyophilized, and analyzed by MALDI-TOF. The purity of the conjugate was confirmed by analytical HPLC (Zorbax 300SB-C18, 3.5 µm particle size, 4.6 × 150 mm, Agilent) and electrospray ionization mass spectrometry (ESI-MS), (Thermo Ultimate3000, Thermo Fisher Scientific, Waltham, MA, USA, + Bruker Impact II, Bruker Daltonics, Billerica, MA, USA) (Figures S7 and S8). 

Final concentrations of the PNA probes and Z_HER2:342_-SR-*HP15* were determined by measuring absorbance at 260 nm. The same extinction coefficient was used for both *HP15* and Z_HER2:342_-SR-*HP15* as the contribution from the protein part to the total absorbance at 260 nm was considered to be negligible. 

The binding of Z_HER2:342_-SR-*HP15* to the HER2 receptor was confirmed by SPR (see supplementary information; Figure S9).

### 4.3. Characterization of the PNA Pretargeting Probes 

The kinetic parameters for hybridization of the PNA probes were analyzed using surface plasmon resonance (SPR) on a Biacore 8K instrument (GE Healthcare). A dextran chip Series S Sensor CM5 (GE Healthcare) was functionalized with Z_HER2:342_-SR-*HP15* on three surfaces to 385, 194, and 185 resonance units (RU) using standard amine coupling procedures. The surface with 385 RU was used for kinetic measurements of *HP16*; 194 RU was used for *HP17*, and 185 RU was used for *HP18*. A reference surface was subjected to activation, followed by deactivation. The complementary PNA probes, *HP16*, *HP17*, and *HP18*, were injected at 5 concentrations—22.6, 45.3, 90.6, 181.25, and 362.5 nM—using a single-cycle injection. Association for each concentration was allowed for 300 s, followed by the next injection and association phase. After injection of the final concentration, dissociation was allowed for 10,000 s (2 h 47 min) before regeneration with 10 mM HCl for 30 s, followed by 15 mM NaOH for 30 s. All runs were performed in PBST (0.05% Tween-20) pH 7.4 using a flow rate of 50 µl/min at 25 °C. Kinetic parameters were calculated using the 1:1 binding model in Biacore Insight Evaluation software. 

Melting temperatures for all three PNA hybridization complexes (*HP15*:*HP16*, *HP15*:*HP17*, and *HP15*:*HP18*) were determined by monitoring UV absorbance at 260 nm (Chirascan, Applied Photophysics) at a temperature range from 20 to 95 °C, using a temperature change of 1 °C/min. The PNA complexes were heated to 80 degrees for 5 min and were then allowed to hybridize at room temperature for 5 min prior to UV monitoring at room temperature for 5 min (Figure 4). CD spectra of PNA:PNA hybridization complexes (Figure S10) were collected before and after the determination of thermal denaturation.

### 4.4. Radiolabeling and In Vitro Stability 

Radiolabeling of the primary and secondary PNA probes with ^177^Lu was performed using a previously described method [21]. Briefly, 30 μg of peptide was dissolved in 100 μL of ascorbic acid (1 M, pH 5.5) by heating at 95 °C for 10 min, followed by sonication for 5 min to ensure total dissolving. Then, 3 μL (60–120 MBq) of [^177^Lu]LuCl_3_ was added, followed by vortexing. The mixture was incubated at 95 °C for 60 min. The reaction mixture was analyzed by radio-ITLC, eluted with 0.2 M citric acid, pH 2.0. 

To remove loosely bound ^177^Lu, a treatment with an excess of ethylenediaminetetraacetic acid tetra sodium salt (EDTANa_4_) was performed initially. A freshly prepared solution of EDTANa_4_ (10 mg/mL in Milli-Q water) was added in 1000-fold molar excess to the reaction mixture and incubated at 95 °C for 10 min. This step was found unnecessary for new secondary probes due to the high radiochemical yield, purity, and stability of the label. [^177^Lu]Lu-*HP16*, [^177^Lu]Lu-*HP17*, and [^177^Lu]Lu-*HP18* were used in biological in vitro and in vivo studies without any additional purification. For [^177^Lu]Lu-*HP2* and [^177^Lu]Lu-Z_HER2:342_-SR-*HP15*, a purification was performed after EDTA treatment by size exclusion chromatography using disposable NAP-5 columns, pre-equilibrated and eluted with 1% BSA/PBS. 

To evaluate stability, a fraction of the freshly radiolabeled conjugate (0.4 µg) was incubated with a 500-fold molar excess of EDTA for 60 min at 37 °C. Incubation was also performed in PBS as a control. The test was run in triplicates.

To validate the results of radio-ITLC, reversed phase-HPLC conducted on an Elite LaChrom system (Hitachi, VWR, Darmstadt, Germany) consisting of an L-2130 pump, a UV detector (L-2400), and a radiation flow detector (Bioscan, Washington, DC, USA) coupled in series was used. Purity analysis of ^177^Lu-labeled compounds was performed using an analytical column (Phenomenex, Aschaffenburg, Germany; Luna^®^ 5 µm C18, 100 Å; 150 × 4.6 mm column). The HPLC conditions were as follows: A = 10 mM TFA/H_2_O; B = 10 mM TFA/acetonitrile; UV-detection at 220 nm; gradient elution: 0–15 min at 5% to 70% B, 15–18 min at 70% to 95% B, 19–20 min at 5% B; flow rate was 1.0 mL/min.

### 4.5. In Vitro Studies

Cells were seeded in cell-culture dishes with a density of 10^6^ cells/dish. A set of three dishes was used for each data point.

The specificity of [^177^Lu]Lu-Z_HER2:342_-SR-*HP15* binding to HER2-expressing cells was tested by the incubation of the cells with 1 nM of labeled conjugate for 1 h at 37 °C. To saturate the receptors, unlabeled Z_HER2:342_ (1000 nM) was added to a control set for 5 min before adding a radiolabeled probe. 

An in vitro pretargeting specificity assay for novel [^177^Lu]Lu-*HP16*, [^177^Lu]Lu-*HP17*, and [^177^Lu]Lu-*HP18* was performed using four sets of cell dishes, as described earlier [16]. To demonstrate the pretargeting, one set of dishes was incubated with Z_HER2:342_-SR-*HP15* (1 nM) for 1 h at 37 °C and washed. A ^177^Lu-labeled secondary probe (10 nM) was added, and cells were incubated for 1 h at 37 °C. To show that the pretargeting was HER2-mediated, the second set of dishes was incubated with an excess of affibody molecules Z_HER2:342_ (1000 nM) for 5 min before adding Z_HER2:342_-SR-*HP15*. A ^177^Lu-labeled secondary probe (10 nM) was added, and cells were incubated for 1 h at 37 °C. To demonstrate that pretargeting was PNA-mediated, the third set of dishes was incubated with Z_HER2:342_-SR-*HP15*, followed by incubation with an excess of a nonlabeled secondary probe (300 nM) for 30 min, and then the ^177^Lu-labeled secondary probe was added, followed by 1 h incubation. In the fourth set, the cells were incubated only with the ^177^Lu-labeled secondary probe to assess nonspecific binding. At the end of the incubation, the cells were washed and detached by trypsin, and the radioactivity in cells was measured to calculate the percent of cell-bound radioactivity.

To evaluate the binding affinity of the radiolabeled conjugates to HER2 receptors and to a cell-bound primary probe, the kinetics of binding of ^177^Lu-labeled probes to, and their dissociation from, SKOV3 cells were measured using a LigandTracer yellow instrument (Ridgeview Instruments AB, Vänge, Sweden). SKOV3 cells (3 × 10^6^ cells/dish) were seeded on a local area of a cell-culture dish (NunclonTM, Size 100620, NUNC A/S, Roskilde, Denmark). The SKOV3 cells were presaturated with Z_HER2:342_-SR-*HP15* (1 nM) in two sets of dishes for 2 h and, thereafter, washed three times to remove the unbound primary agent. Two increasing concentrations of the radiolabeled molecules (for [^177^Lu]Lu-Z_HER2:342_-SR-*HP15*: 180 and 540 pM, and for ^177^Lu-labeled secondary probes: 1 and 5 nM) were added. The data was analyzed using Interaction Map software (Ridgeview Diagnostics AB, Uppsala, Sweden) to calculate the association rate constant (k_a_), the dissociation rate constant (k_d_), and the equilibrium dissociation constant (K_D_). The analysis was performed in duplicate.

Cellular processing and retention on SKOV3 and BT474 cells were studied during interrupted incubation by an acid-wash method [14]. 

### 4.6. In Vivo Studies 

Animal studies were planned in agreement with EU Directive 2010/63/EU for animal experiments and Swedish national legislation concerning the protection of laboratory animals and were approved by the Ethics Committee for Animal Research in Uppsala, Sweden (animal permission C4/16). For tumor implantation, 10^7^ SKOV3 cells were subcutaneously injected on the right hind leg of female BALB/c nu/nu mice. The biodistribution experiments were performed two weeks after cell implantation. The average animal weight was 18 ± 1 g. The average tumor weight was 0.23 ± 0.11 g. For biodistribution measurement, the mice were euthanized at predetermined time points by an overdose of anesthesia (ketamine/xylazine), followed by heart puncture. The organs of interest and the tumor were collected and weighed, and their radioactivity was measured. The percentage of the total injected dose per gram of sample (% ID/g) was calculated. 

The pretargeting protocol used in this study has previously been optimized for the affibody-based PNA-mediated therapy using [^177^Lu]Lu-*HP2* [21]. Upscaling experiments showed no significant changes in the biodistribution of [^177^Lu]Lu-*HP2* when the injected mass of both primary and [^177^Lu]Lu-*HP2* was doubled. Based on this study, 50 µg of primary agents and equimolar amounts of secondary agents (0.69, 0.85, 1, and 1 µg for *HP16*, *HP17*, *HP18*, and *HP2*, respectively) were injected per mouse. 

For biodistribution studies, mice were randomized into groups of five. The 30 mice were intravenously injected with Z_HER2:342_-SR-*HP15* (50 µg, 4 nmol in 100 μL PBS per mouse). Sixteen hours later, all mice were injected with [^177^Lu]Lu-*HP16*, [^177^Lu]Lu-*HP17*, or [^177^Lu]Lu-*HP18* (194 pmol in 100 μL 2% BSA in PBS, 170 kBq). The biodistribution was measured at 4 and 144 h after injection of secondary probes. For comparison, the biodistribution of first-generation [^177^Lu]Lu-*HP2* was measured in the same way using Z_HER2:342_-SR-*HP1* as the primary agent. 

To evaluate in vivo specificity, the biodistribution of [^177^Lu]Lu secondary probes was measured 4 h after injection, without the preinjection of a primary agent. 

After the gamma-counter measurements were completed, tumors were embedded in a cryomedium (Neg-50, Thermo Scientific) and frozen at −80 °C. Frozen tumors were cut into serial sections (30 µm thick) using a cryomicrotome (CryoStar NX70, Thermo Scientific) and thaw-mounted on glass slides. For digital autoradiography, the slides with sections were put in a cassette and exposed to phosphor screens overnight. The phosphor screens were scanned by a Cyclone Storage Phosphor System at 600 dpi resolution and analyzed using OptiQuant software (PerkinElmer, Waltham, MA, USA).

To estimate a ratio of absorbed doses in tumor and kidneys, cumulated activity in kidney and tumor were assessed. The estimation was based on a clinically validated two-time point approach [26]. The biodistribution data were nondecay-corrected, and areas under the time-activity plot were calculated using GraphPad Prism software. The assumptions were that the main absorbed dose would be due to betaparticles, as cross-doses would be negligible, and the absorbed fraction would be equal to 1. 

### 4.7. SPECT/CT Imaging 

Mice bearing SKOV3 xenografts were injected i.v. with 8 nmol of primary agent 16 h before injection of secondary ^177^Lu-labeled probes (680 pmol, 9–13 MBq). Immediately before imaging (4 h after injection), the animals were sacrificed by CO_2_ asphyxiation. SPECT imaging was performed using NanoScan SC (Mediso Medical Imaging Systems, Budapest, Hungary). CT acquisitions were carried out using the X-ray energy of 50 keV; 20-min SPECT helical scans were acquired using energy windows 50–62, 103–124, and 188–230 keV. The data were reconstructed using Tera-Tomo™ 3D SPECT Software.

## 5. Conclusions

In conclusion, the molecular design of the second-generation hybridization probes presented here has provided a primary agent Z_HER2:342_-SR-*HP15* that binds with high specificity and high affinity to HER2-expressing cells in vitro and a set of secondary PNA-based hybridization probes that bind with high affinity to the primary agent. The difference in the number of nucleobases in the secondary probes, in the range between 9 and 15, apparently had no relevant influence on the strength of their binding to primary probes in the cell assay, although a difference was observed in the SPR and UV analyses. The secondary probes demonstrated different biodistribution properties, and the best pretargeting was provided by [^177^Lu]Lu-*HP16*, which has a 9-mer PNA sequence.

## Figures and Tables

**Table 1:** Peptide nucleic acid (PNA) probe sequences. Amino acids are denoted by uppercase letters, and PNA monomers are denoted by lowercase letters. DOTA is the chelator 1,4,7,10-tetraazacyclododecane-1,4,7,10-tetraacetic acid, and AEEA is the PEG-based linker NH_2_-(PEG)_2_-CH_2_COOH (also referred to as 2-[2-(2-aminoethoxy)ethoxy]acetic acid).

Name	Sequence	Reference	Mw [Da]
*HP1*	G-G-G-S-S-a-g-t-c-t-g-g-a-t-g-t-a-g-t-c-E-K(DOTA)-AEEA-E-NH_2_	[20]	5396
*HP2*	DOTA-AEEA-S-S-g-a-c-t-a-c-a-t-c-c-a-g-a-c-t-E-E-Y-NH_2_	[20]	5158
*HP15*	G-S-S-c-c-t-g-g-t-g-t-t-g-a-t-g-a-t-E-K(DOTA)-AEEA-E-NH_2_	New	5273
*HP16*	DOTA-AEEA-S-S-a-a-c-a-c-c-a-g-g-E-E-Y-NH_2_	New	3582
*HP17*	DOTA-AEEA-S-S -a-t-c-a-a-c-a-c-c-a-g-g-E-E-Y-NH_2_	New	4375
*HP18*	DOTA-AEEA-S-S-a-t-c-a-t-c-a-a-c-a-c-c-a-g-g-E-E-Y-NH_2_	New	5167

**Table 2:** Kinetic parameters and melting temperatures for the secondary PNA probes *HP16*, *HP17*, and *HP18* binding to Z_HER2:342_-SR-*HP15*.

Construct	K_D_ (pM)	k_a_ (M^−1^ s^−1^)	k_d_ (s^−1^)	Tm (°C)
*HP16*	280	4.6 × 10^4^	1.2 × 10^−5^	73
*HP17*	n.d.	4.3 × 10^4^	n.d.	75
*HP18*	n.d.	5.7 × 10^4^	n.d.	87

n.d. = not determined.

**Table 3:** ^177^Lu-labeling of PNA-based probes and in vitro stability.

Construct	Radiochemical Yield, %	Radiochemical Yield after EDTA Treatment, %	Isolated Yield, %	Radiochemical Purity, %	Stability (×500 EDTA), 37 °C, 1 h
Z_HER2:342_-SR-*HP15*	78.5 ± 6.4	65.0 ± 7.1	66.0 ± 1.4	100 ± 0	100 ± 0
*HP16*	99.8 ± 0.3	99.2 ± 1.4	-	99.2 ± 1.4	100 ± 0
*HP17*	99.0 ± 1.7	98.8 ± 2.0	-	98.8 ± 2.0	100 ± 0
*HP18*	97.5 ± 2.0	98.4 ± 2.7	-	98.4 ± 2.7	100 ± 0
*HP2*	73.2 ± 0.2	69.6 ± 2.6	51.5 ± 9.2	100 ± 0	-

**Table 4:** Biodistribution of new ^177^Lu-labeled secondary probes in BALB/C nu/nu mice bearing SKOV3 xenografts at 4 h p.i. without preinjection of a primary agent. The uptake is expressed as % ID/g and presented as an average value from 5 mice ± SD. One-way ANOVA with Bonferroni’s multiple comparisons test was performed to find significant differences.

	Uptake, % ID/g		
Organ or Tissue	[^177^Lu]-*HP16*	[^177^Lu]-*HP17*	[^177^Lu]-*HP18*
Blood	0.007 ± 0.003	0.005 ± 0.001 ^c^	0.019 ± 0.006 ^b^
Lung	0.09 ± 0.02	0.07 ± 0.01 ^c^	0.12 ± 0.02
Liver	0.08 ± 0.01	0.08 ± 0.02 ^c^	0.11 ± 0.02 ^b^
Spleen	0.053 ± 0.008	0.06 ± 0.01	0.08 ± 0.02
Kidney	3.6 ± 0.6	4.5 ± 0.4 ^c^	9.1 ± 0.9 ^b^
Tumor	0.12 ± 0.03	0.08 ± 0.01	0.10 ± 0.01
Muscle	0.025 ± 0.004	0.023 ± 0.006	0.029 ± 0.003
Bone	0.051 ± 0.008 ^a^	0.036 ± 0.007 ^c^	0.07 ± 0.02
GI *	0.29 ± 0.06	0.86 ± 0.21	0.65 ± 0.34
Carcass *	1.0 ± 0.4	1.18 ± 0.43	1.17 ± 0.17

^a^ Significant difference between [^177^Lu]Lu-*HP16* and [^177^Lu]Lu-*HP17*; ^b^ significant difference between [^177^Lu]Lu-*HP16* and [^177^Lu]Lu-*HP18*; ^c^ significant difference between [^177^Lu]Lu-*HP17* and [^177^Lu]Lu-*HP18*; * data for the GI tract, with content and carcass, are presented as % of injected dose per whole sample.

**Table 5:** Biodistribution comparison of new ^177^Lu-labeled secondary probes in BALB/C nu/nu mice bearing SKOV3 xenografts at 4 and 144 h after injection. Primary agents (Z_HER2:342_-SR-*HP1* and Z_HER2:342_-SR-*HP15*, 50 µg) were injected 16 h prior to ^177^Lu-labeled secondary probe injection. The uptake is expressed as % ID/g and presented as an average value from 5 mice ± SD. One-way ANOVA with Bonferroni’s multiple comparisons test was performed to find significant differences.

Site	Uptake, % ID/g							
	4 h				144 h			
	*HP16*	*HP17*	*HP18*	*HP2*	*HP16*	*HP17*	*HP18*	*HP2*
Blood	0.04 ± 0.01 ^a^	0.05 ± 0.01 ^b^	0.17 ± 0.02 ^a,b,c^	0.049 ± 0.004 ^c^	NM	NM	NM	NM
Lung	0.16 ± 0.03 ^d^	0.17 ± 0.04 ^b^	0.30 ± 0.03 ^b,c,d^	0.19 ± 0.03^c^	NM	NM	NM	NM
Liver	0.10 ± 0.02 ^a^	0.11 ± 0.01 ^b^	0.2 ± 0.1 ^a,b,c^	0.11 ± 0.02 ^c^	0.014± 0.003 ^a^	0.010 ± 0.004 ^b^	0.07 ± 0.01 ^a,b,c^	0.015 ± 0.003 ^c^
Spleen	0.07 ± 0.01 ^a^	0.09 ± 0.03	0.12 ± 0.02 ^a^	0.08± 0.03	NM	NM	NM	NM
Kidney	6 ± 1 ^a,d^	8 ± 1 ^b^	12 ± 2 ^a,b^	10 ± 2 ^d^	0.7 ± 0.2 ^a^	0.67 ± 0.05 ^b^	1.6 ± 0.5 ^a,b,c^	0.9 ± 0.2 ^c^
Tumor	24 ± 6	22 ± 3	22 ± 1	19 ± 1	3 ± 1 ^a^	5 ± 1	5 ± 1 ^a^	4 ± 1
Muscle	0.06 ± 0.02	0.06 ± 0.02	0.07 ± 0.03	0.04 ± 0.01	NM	NM	NM	NM
Bone	0.06 ± 0.02 ^a^	0.07± 0.01	0.10 ± 0.02 ^a,c^	0.08 ± 0.06 ^c^	NM	NM	NM	NM
GI	0.4 ± 0.1	0.5 ± 0.3	0.4 ± 0.2	0.4 ± 0.1	0.02 ± 0.01	0.05 ± 0.02	0.08 ± 0.06	0.09± 0.07
Carcass	0.7 ± 0.1	1.5± 0.9	1.8± 0.9	1.6 ± 0.7	0.09 ± 0.02	0.12 ± 0.07	0.24 ± 0.02	0.2 ± 0.1

NM = not measurable. ^a^ Significant difference (*p* < 0.05) between [^177^Lu]Lu-*HP16* and [^177^Lu]Lu-*HP18*; ^b^ significant difference (*p* < 0.05) between [^177^Lu]Lu-*HP1*7 and [^177^Lu]Lu-*HP18*; ^c^ significant difference (*p* < 0.05) between [^177^Lu]Lu-*HP18* and [^177^Lu]Lu-*HP2*; ^d^ significant difference (*p* < 0.05) between [^177^Lu]Lu-*HP16* and [^177^Lu]Lu-*HP2*.

**Table 6:** Comparison of tumor-to-kidney ratios of new ^177^Lu-labeled secondary probes in BALB/C nu/nu mice bearing SKOV3 xenografts at 4 and 144 h after injection. Primary agents (Z_HER2:342_-SR-*HP1* and Z_HER2:342_-SR-*HP15*, 50 µg) were injected 16 h prior to ^177^Lu-labeled-secondary probe injection. The uptake is expressed as % ID/g and presented as an average value from 5 mice ± SD. One-way ANOVA with Bonferroni’s multiple comparisons test was performed to find significant differences.

	Tumor-to-Kidney Ratio			
Time	*HP16*	*HP17*	*HP18*	*HP2*
4 h	3.9 ± 0.9	2.7 ± 0.5 ^a^	1.9 ± 0.4 ^b^	2.0 ± 0.4 ^c^
144 h	5 ± 1	7 ± 2 ^d^	3 ± 1	4.2 ± 0.6

^a^ Significant difference (*p* < 0.05) between [^177^Lu]Lu-*HP16* and [^177^Lu]Lu-*HP17*; ^b^ highly significant difference (*p* < 0.0005) between [^177^Lu]Lu-*HP16* and [^177^Lu]Lu-*HP18*; ^c^ highly significant difference (*p* < 0.0005) between [^177^Lu]Lu-*HP16* and [^177^Lu]Lu-*HP2*; ^d^ highly significant difference (*p* < 0.001) between [^177^Lu]Lu-*HP17* and [^177^Lu]Lu-*HP18*.

## Data Availability

The data generated during the current study are available from the corresponding author upon reasonable request.

## Supplementary Materials

The following are available online at https://www.mdpi.com/2072-6694/13/3/500/s1. Figure S1: Analytical RP-HPLC chromatograms of the purified PNA probes *HP16*, *HP17*, and *HP18*. Figures S2–S4: MALDI-TOF spectra of the purified PNA probes *HP16*, *HP17*, and *HP18*. Figure S5: SDS-PAGE gel of Z_HER2:342_-SR-H_6_. Figure S6: MALDI-TOF spectra of purified Z_HER2:342_-SR-H_6_. Figure S7: RP-HPLC chromatograms of sortase A conjugation reaction and purified Z_HER2:342_-SR-*HP15*. Figure S8: LC-MS analysis of purified Z_HER2:342_-SR-*HP15*. Figure S9: SPR sensorgram of Z_HER2:342_-SR-*HP15:HP18* binding to immobilized HER2-Fc. Figure S10: CD spectra of *HP15:HP16*, *HP15:HP17*, and *HP15:HP18*. Figure S11: Distribution of radioactivity of [^177^Lu]Lu-*HP16*, [^177^Lu]Lu-*HP17*, and [^177^Lu]Lu-*HP18* along the ITLC strip. Figure S12: RP-HPLC chromatograms of nonlabeled probes and radiochromatograms of [^177^Lu]Lu-*HP16*, [^177^Lu]Lu-*HP17*, and [^177^Lu]Lu-*HP18*. Figure S13: Interaction map of complexes binding to HER2-expressing SKOV3 cells. Figure S14: Cellular processing and retention by SKOV3 and BT474 cells after interrupted incubation with labeled compounds. Figure S15: Ex vivo autoradiography of tumor slices from the pretargeting experiment 4 h after injection. Figure S16: Ex vivo autoradiography of tumor slices from the pretargeting experiment 144 h after injection. Figure S17: Time-activity plots for [^177^Lu]Lu-*HP16*, [^177^Lu]Lu-*HP17*, [^177^Lu]Lu-*HP18*, and [^177^Lu]Lu-*HP2*.
