# Supplementary material for: Comparative Evaluation of Novel 177Lu-Labeled PNA Probes for Affibody-Mediated PNA-Based Pretargeting
Source: Cancers (Basel). 2021 Jan 28;13(3):500. doi: 10.3390/cancers13030500 (PMC7865858; doi:10.3390/cancers13030500)
Supplement: Supplementary file 1 [file cancers-13-00500-s001.pdf]

Comparative Evaluation of Novel [^{177}Lu]Lu-labeled PNA Probes for Affibody-Mediated PNA-based Pretargeting

Hanna Tano, Maryam Oroujeni, Anzhelika Vorobyeva, Kristina Westerlund, Yongsheng Liu, Tianqi Xu, Daniel Vasconcelos, Anna Orlova, Amelie Eriksson Karlström and Vladimir Tolmachev

Supplementary Information

Production and purification of PNA-based pretargeting agents

Purity of secondary PNA probes were estimated to >90% according to RP-HPLC (Figure S1) and MALDI-TOF/TOF (Figures S2–S4).

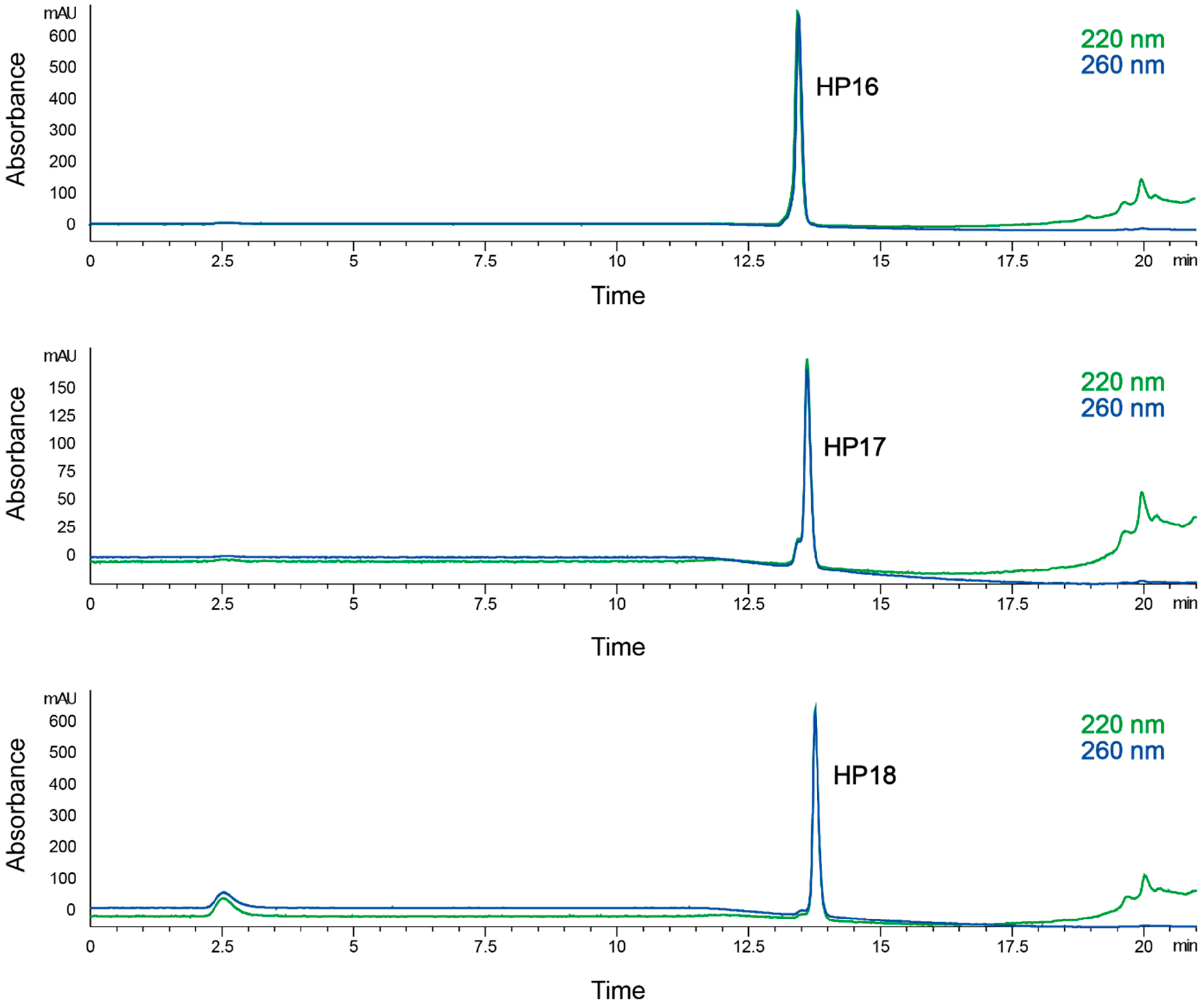

Figure S1. Analytical RP-HPLC chromatograms of the purified PNA probes *HP16*, *HP17* and *HP18* monitored at 220 nm (green line) and 260 nm (blue line). Peaks at 220 nm after 20 min (at 100% B: 0.1% TFA in CH_3CN) were found also in blank injections.

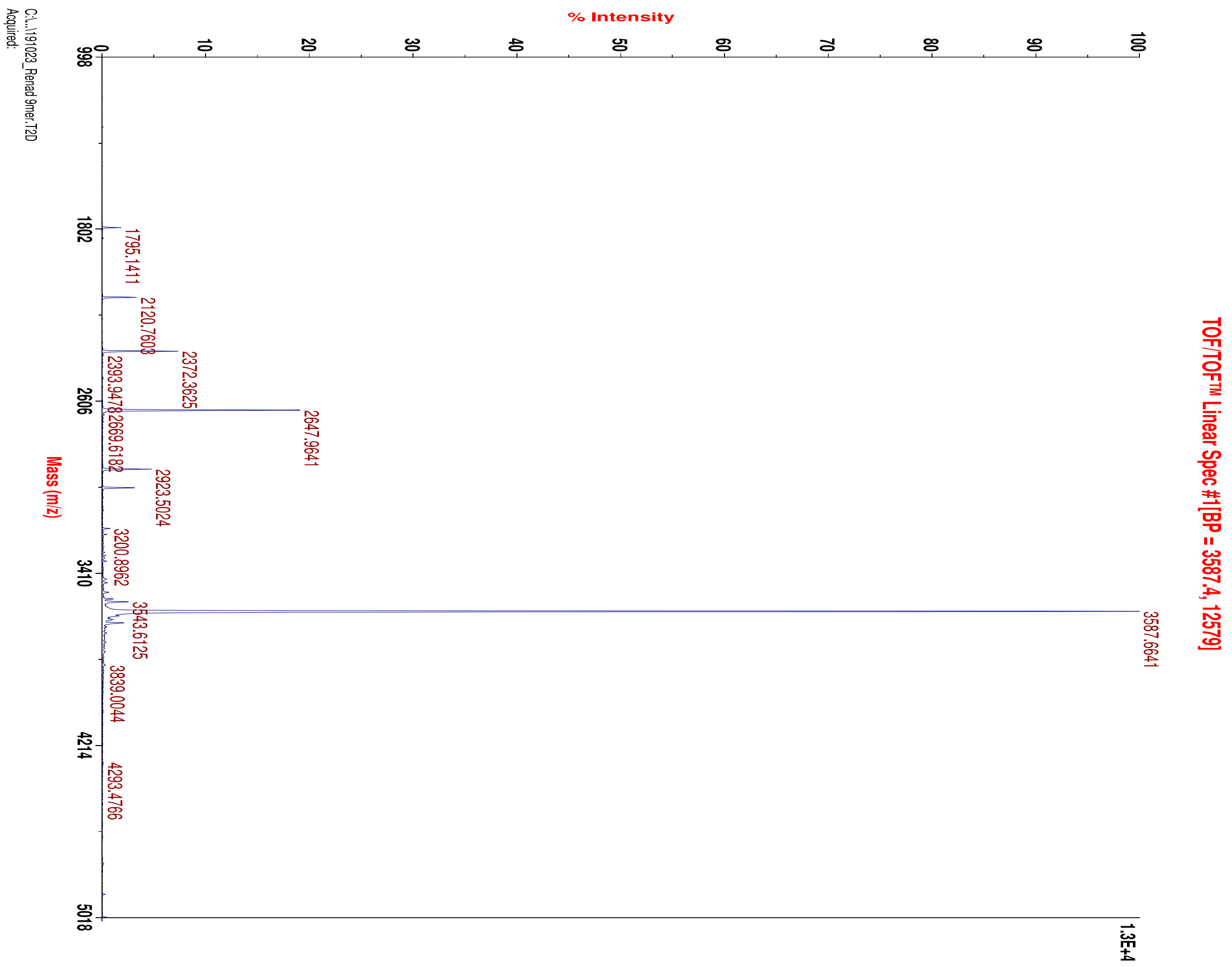

Figure S2. MALDI-TOF spectra of purified PNA probe *HP16*. Observed molecular weight is 3588 Da, theoretical molecular weight is 3582 Da.

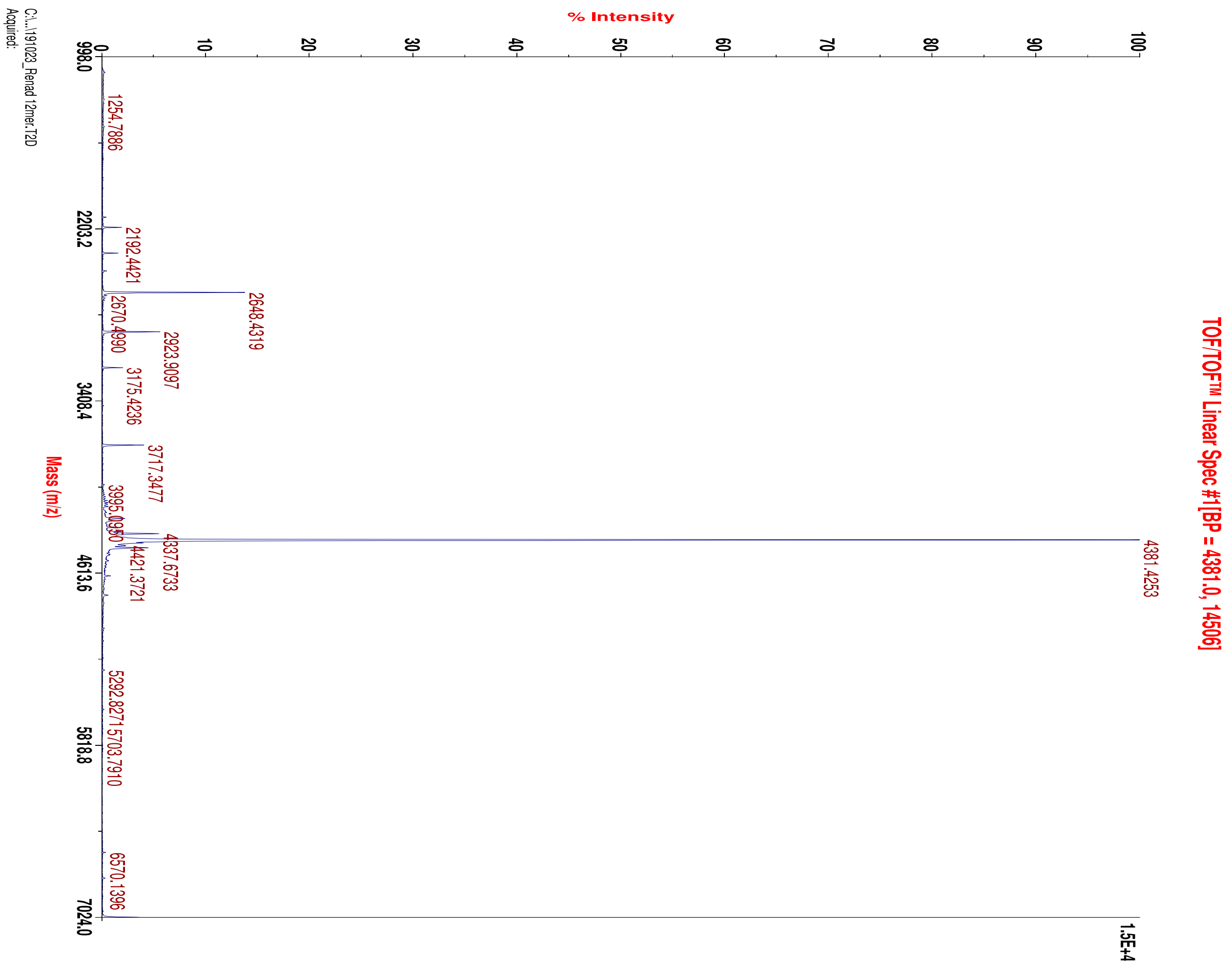

Figure S3. MALDI-TOF spectra of purified PNA probe *HP17*. Observed molecular weight is 4381 Da, theoretical weight is 4375 Da.

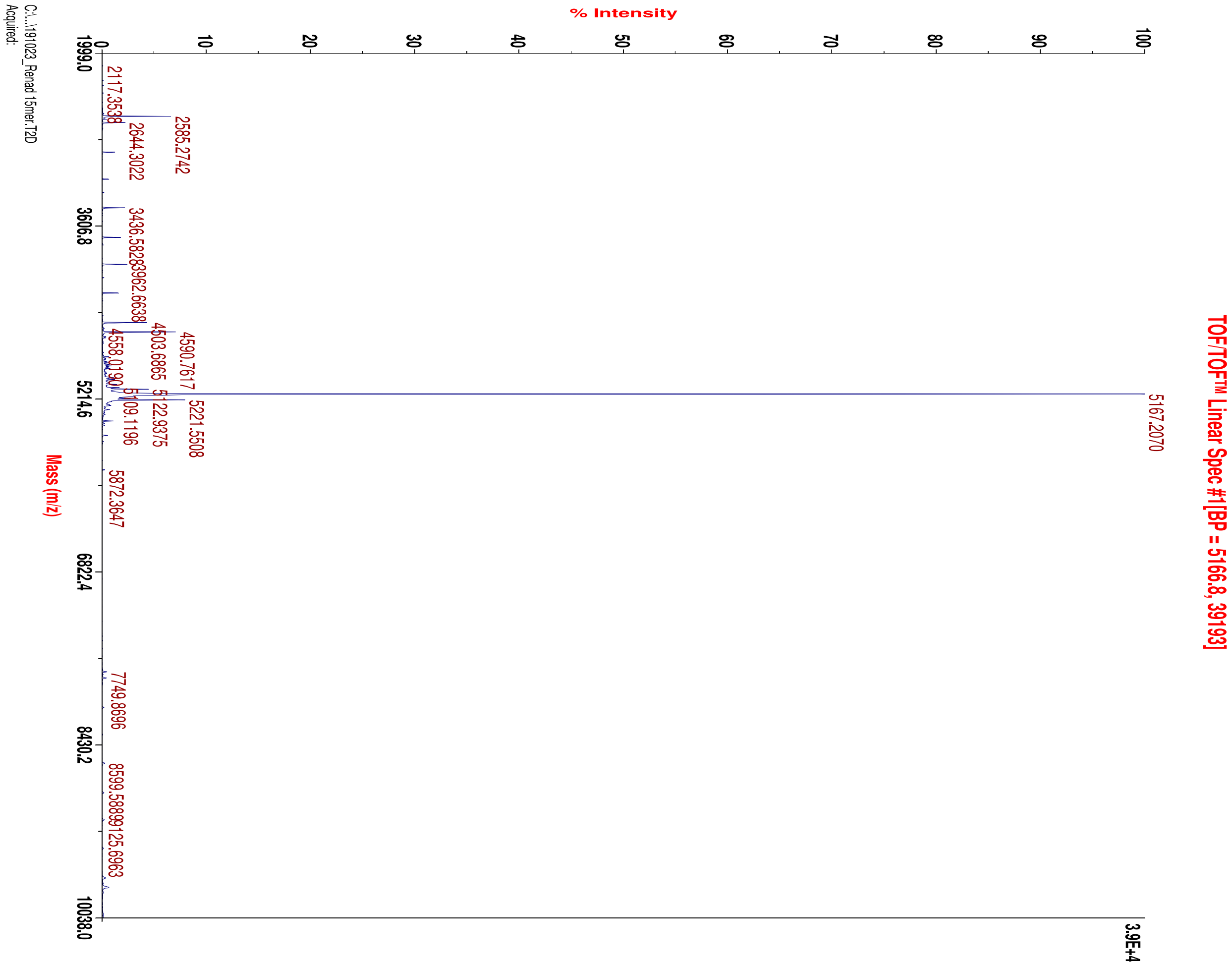

Figure S4. MALDI-TOF spectra of purified PNA probe *HP18*. Observed molecular weight is 5167 Da, theoretical molecular weight is 5167 Da.

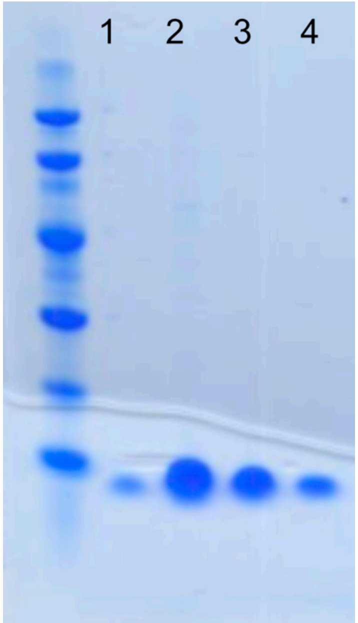

Figure S5. SDS-PAGE gel of 4 purified samples (lanes 1-4) of $Z_{HER2:342}$-SR-H_6 (10 µl loaded of each sample, with protein concentrations ranging from ca. 0.1 µg/µl to ca. 1 µg/µl). Ladder: 14.4, 20.1, 30, 45, 66 and 97 kDa (5 µl loaded of Amersham Low Molecular Weight Calibration for SDS Electrophoresis, GE Healthcare).

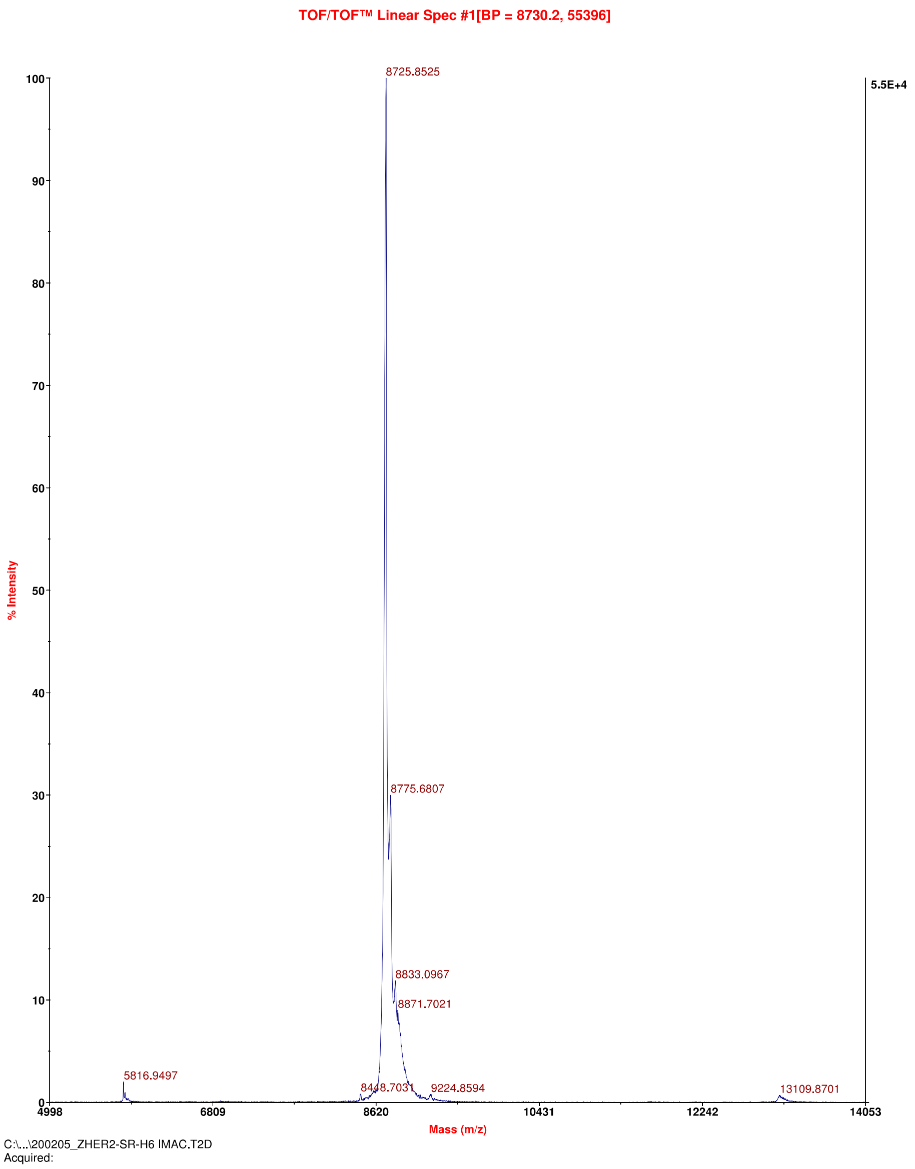

Figure S6. MALDI-TOF spectra of purified $Z_{HER2:342}$-SR-H_6. Observed molecular weight is 8726 Da, theoretical molecular weight is 8730 Da.

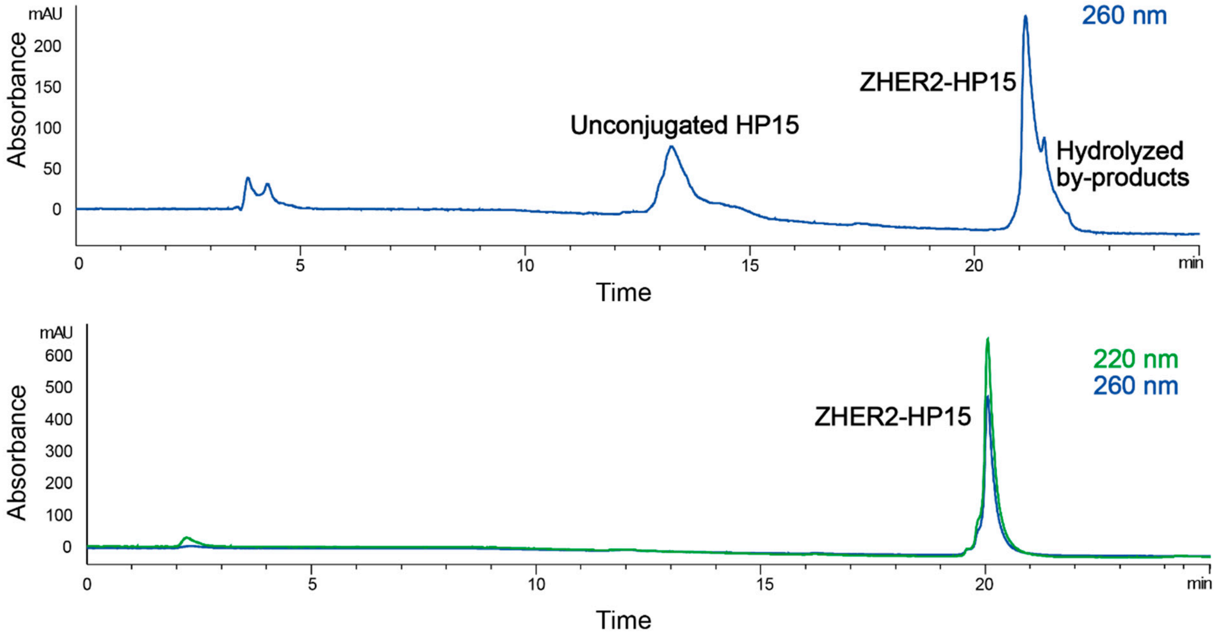

Figure S7. RP-HPLC chromatograms monitored at 220 nm (green line) and 260 nm (blue line). Top: Sortase A conjugation reaction of $Z_{HER2:342}$-SR-H_6 and *HP15*. This representative chromatogram was used to estimate the conjugation efficiency (~40%). Bottom: purified $Z_{HER2:342}$-SR-*HP15*.

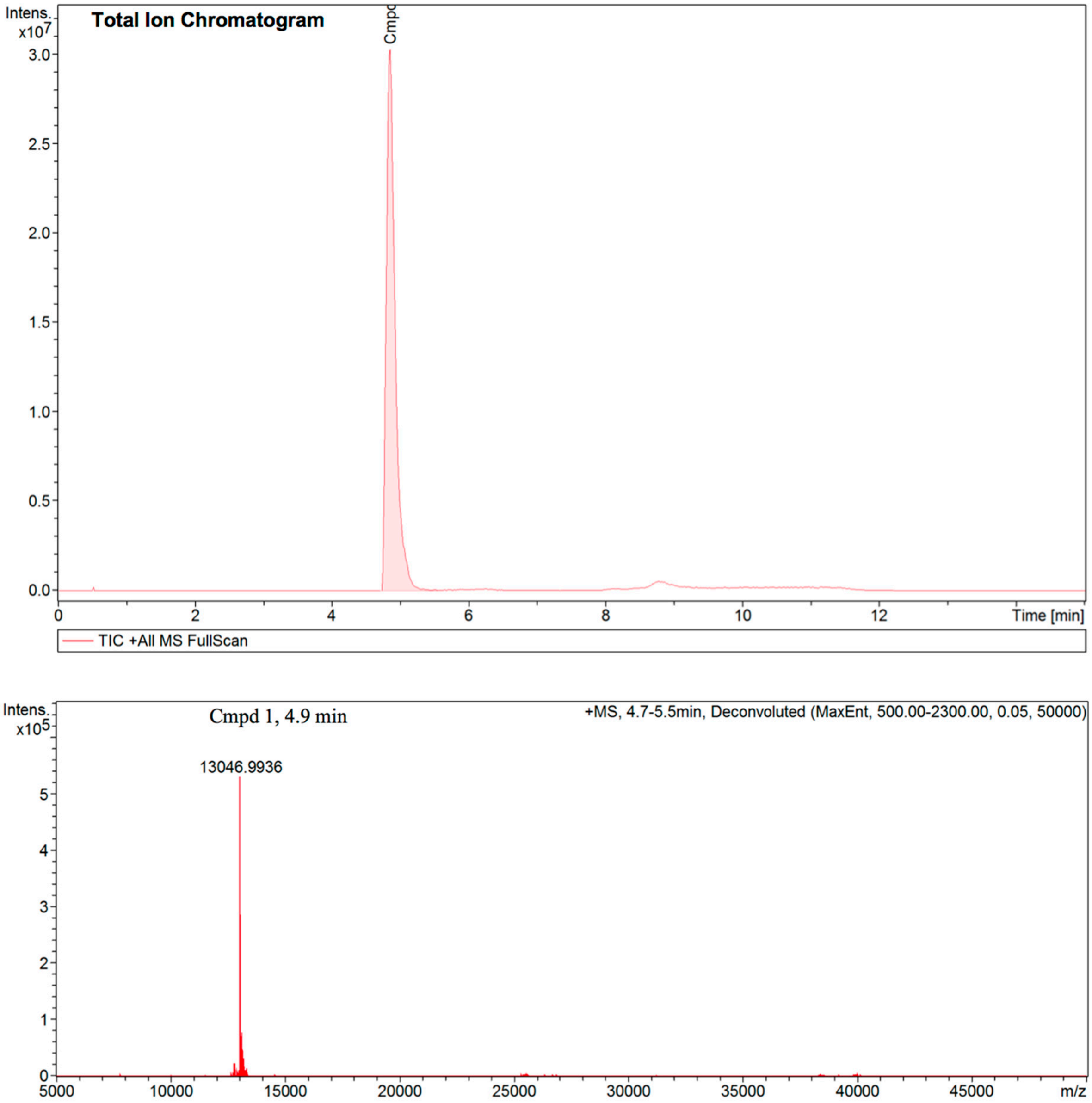

Figure S8. LC-MS analysis of purified $Z_{HER2:342}$-SR-*HP15*. The purity is estimated to >95% and the observed molecular weight is 13047 Da.

Characterization of the affibody-PNA conjugate and the complementary PNA probes

In order to verify simultaneous binding of $Z_{HER2:342}$-SR-*HP15* to *HP18* and the HER2 receptor, HER2-Fc (Sino Biological) was immobilized to a dextran chip Series S Sensor CM5 at 757 RU. PNA probe *HP18* and $Z_{HER2:342}$-SR-*HP15* were mixed at a 1:1 concentration ratio to allow for hybridization prior to injection to HER2-Fc. Pre-hybridized $Z_{HER2:342}$-SR-*HP15:HP18* were injected at 6 concentrations; 0.78, 1.56, 3.13, 6.25, 12.5 and 25 nM (Figure S9). Association was allowed for 300 s and dissociation was allowed for 2400 s (40 min), followed by regeneration by injection of 20 mM HCl for 20 s. For the highest injected concentration (25 nM), a dissociation time of 7200 s (2 h) was used. All runs were performed in PBST (0.05 % Tween-20) pH 7.4 using a flow rate of 50 µl/min at 25 °C. After regeneration with HCl, running buffer was passed over the sensor surface for 2 h before injection of the next sample.

The equilibrium dissociation constant (K_D) for $Z_{HER2:342}$-SR-*HP15:HP18* binding to HER2-Fc was calculated to 276 pM, with an association rate constant (k_a) of 2.5 x 10^6 $M^{-1}s^{-1}$ and a dissociation rate constant (k_d) of 7.0 x 10^{-4} s^{-1}. This compares well with previously reported kinetic constants for $Z_{HER2:342}$-SR-*HP1:HP2* binding to HER2, where K_D was estimated to 212 pM, with the association rate constant k_a= 1.4 x 10^6 $M^{-1}s^{-1}$ and the dissociation rate constant k_d=2.9 x 10^{-4} s^{-1}.[1] The K_D for the PNA-conjugated affibodies is approximately 10-fold higher than the K_D for unmodified $Z_{HER2:342}$, which has been reported to be 22 pM.[2] This supports the idea that PNA conjugation only marginally interferes with $Z_{HER2:342}$ binding to HER2.

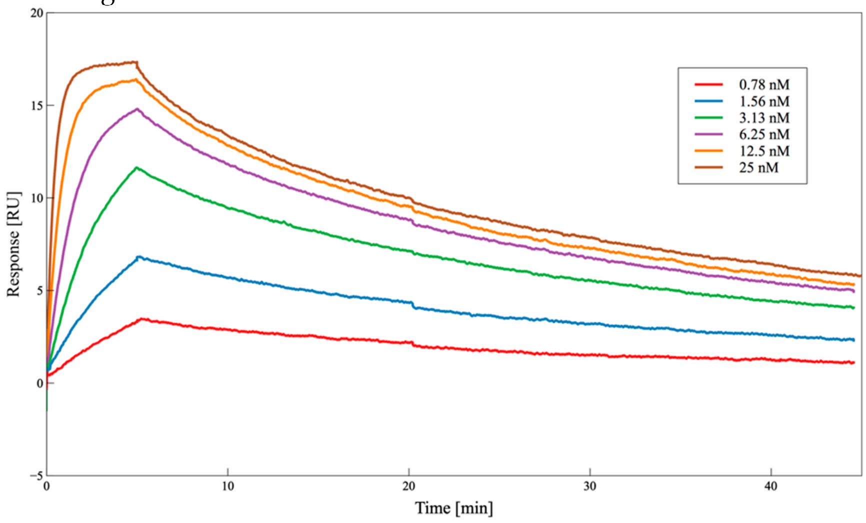

Figure S9. SPR sensorgram of $Z_{HER2:342}$-SR-*HP15:HP18* binding to immobilized HER2-Fc. Pre-hybridized $Z_{HER2:342}$-SR-*HP15:HP18* was injected at concentrations 0.78, 1.56, 3.13, 6.25, 12.5 and 25 nM.

CD spectroscopy (Chirascan, Applied Photophysics) was used to investigate the secondary structure of the PNA:PNA hybridization complexes. CD signal was recorded at wavelengths ranging from 195 to 300 nm. All CD spectra were recorded at a protein/PNA concentration of 0.15–0.2 mg/ml in 20 mM potassium phosphate buffer with 100 mM KCl at pH 7.4.

The hybridization between *HP15* and the secondary probes *H16*, *HP17* or *HP18* gave rise to CD spectra with minima at approximately 215 nm and 260 nm (Figure S10). The induced signals are the result of PNA:PNA double helix formation upon hybridization between complementary PNA probes carrying C-terminal L-amino acids.

[1] *Design, Preparation, and Characterization of PNA-Based Hybridization Probes for Affibody-Molecule-Mediated Pretargeting* , Kristina Westerlund, Hadis Honarvar, Vladimir Tolmachev, and Amelie Eriksson Karlström

[2] Orlova, A., Magnusson, M., Eriksson, T. L., Nilsson, M., Larsson, B., Höideń Guthenberg, I., Widström, C., Carlsson, J., Tolmachev, V., Ståhl, S., and Nilsson,F.Y. (2006) *Tumor imaging using a picomolar affinity HER2 binding affibody molecule.* Cancer Res. 66, 4339−4348

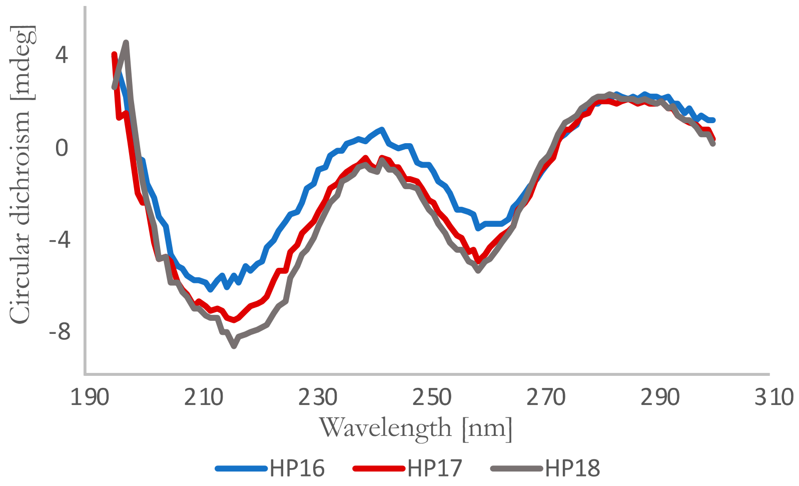

Figure S10. CD spectra of *HP15:HP16* (blue line), *HP15:HP17* (red line) and *HP15:HP18* (grey line).

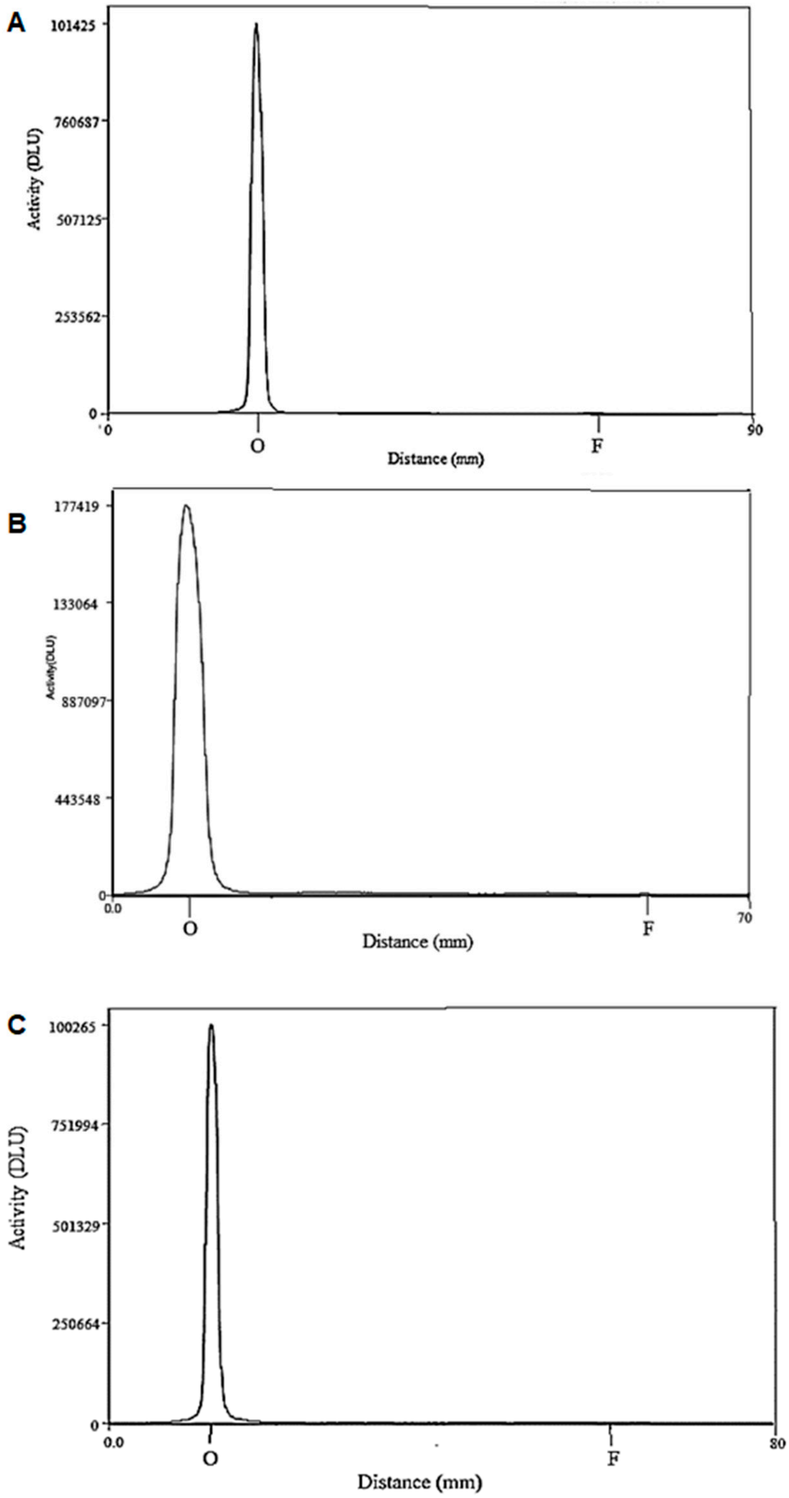

Figure S11. Distribution of radioactivity of (**A**) $[^{177}Lu]Lu$-*HP16*, (**B**) $[^{177}Lu]Lu$-*HP17* and (**C**) $[^{177}Lu]Lu$-*HP18* along an ITLC strip. Retardation factor of labeled conjugate is 0.0 and that of free ^{177}Lu is 1.0.

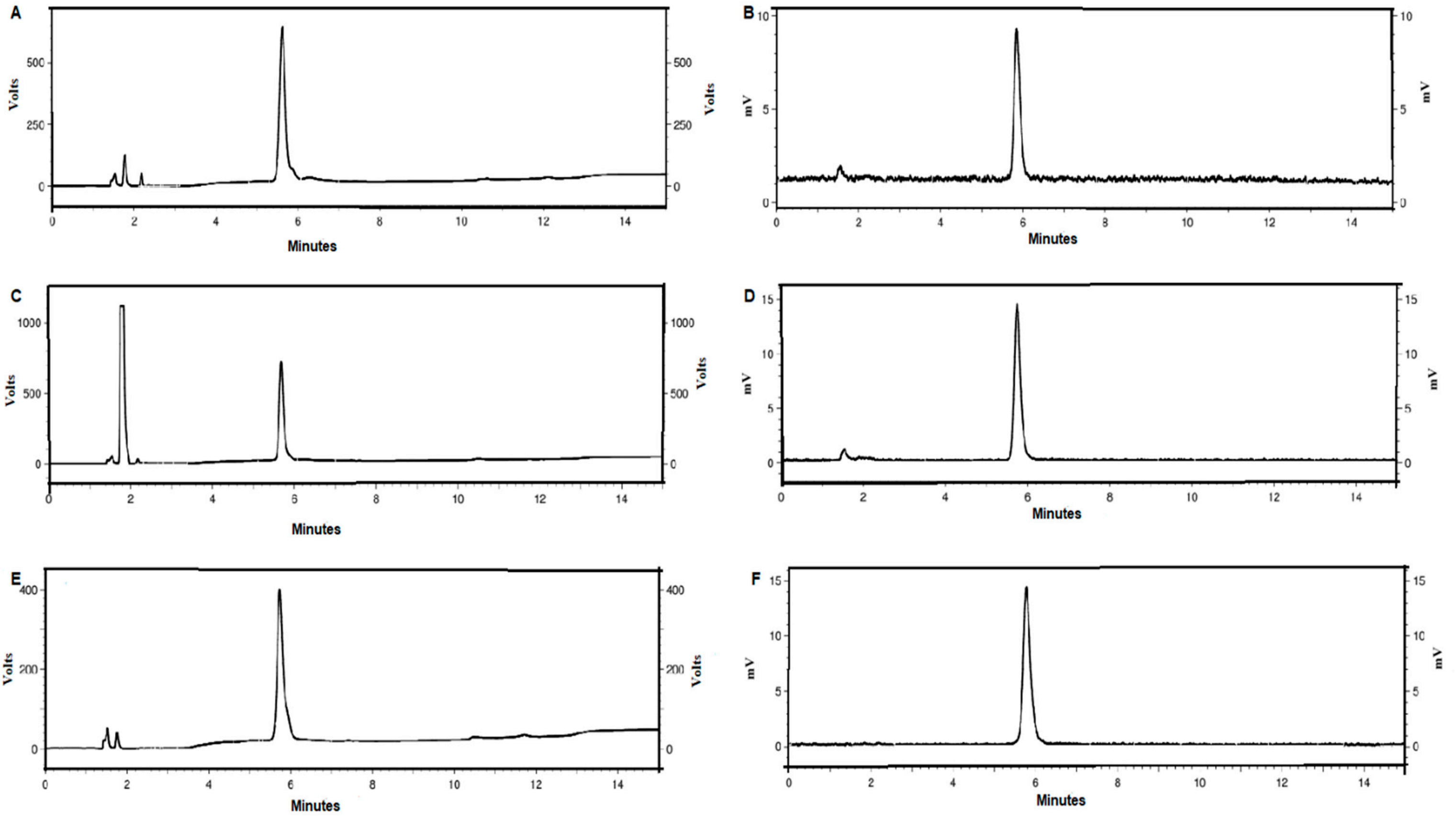

Figure S12. Characterization of secondary probes. Reversed-phase HPLC chromatograms of non-labeled (**A**) *HP16*, (**C**) *HP17* and (**E**) *HP18*; and the radiochromatograms of (**B**) [^{177}Lu]Lu-*HP16*, (**D**) [^{177}Lu]Lu-*HP17* and (**F**) [^{177}Lu]Lu-*HP18*.The retention times (Rt) are expressed in minutes.

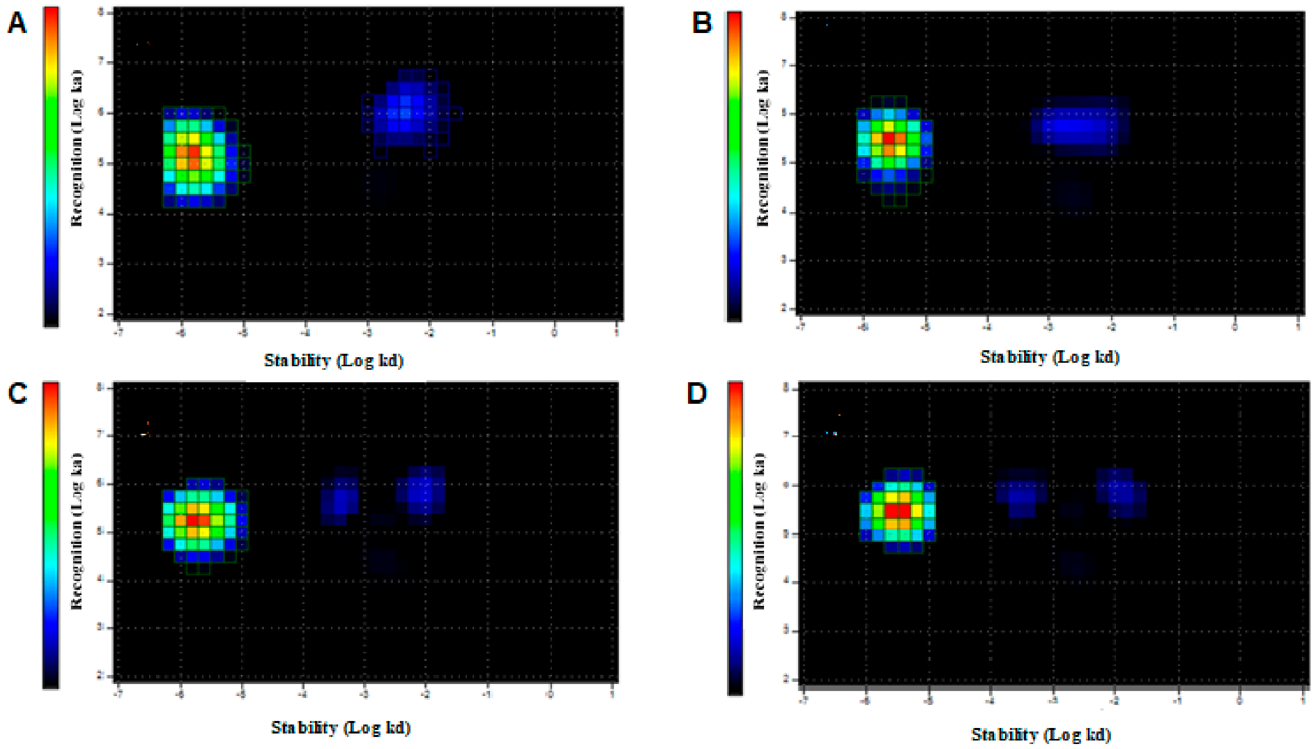

Figure S13. Interaction Map of (**A**) [^{177}Lu]Lu-$Z_{HER2:342}$-SR-*HP15*, (**B**) $Z_{HER2:342}$-SR-*HP15* + [^{177}Lu]Lu-$Z_{HER2:342}$-SR-*HP16* (**C**) $Z_{HER2:342}$-SR-*HP15* + [^{177}Lu]Lu-$Z_{HER2:342}$-SR-*HP17* and (**D**) $Z_{HER2:342}$-SR-*HP15* + [^{177}Lu]Lu-$Z_{HER2:342}$-SR-*HP18* complexes binding to HER2-expressing SKOV3 cells. Binding was measured at two different concentrations: [^{177}Lu]Lu-$Z_{HER2:342}$-SR-*HP15* (180, 540 pM), $Z_{HER2:342}$-SR-*HP15* (1 nM) and [^{177}Lu]Lu-secondary probes (1, 5 nM).

Table S1. Apparent equilibrium dissociation (K_D) constants for the interaction between [^{177}Lu]-Lu-PNA probes and HER2-expressing SKOV3 cells determined using an Interaction Map analysis of the LigandTracer sensorgrams.

Probe	K_a (1/M×s))×10^5	K_d (1/s) ×10^{-6}	K_D (pM)
[^{177}Lu]Lu-$Z_{HER2:342}$-SR-*HP15*	1.5 ± 0.3	1.7 ± 0.0	11.2 ± 1.9
$Z_{HER2:342}$-SR-*HP15* + [^{177}Lu]Lu-*HP16*	2.6 ± 0.4	2.9 ± 0.2	11.4±0.4
$Z_{HER2:342}$-SR-*HP15* + [^{177}Lu]Lu-*HP17*	1.7 ±0.0	2.1 ±0.0	12.1±0.2
$Z_{HER2:342}$-SR-*HP15* + [^{177}Lu]Lu-*HP18*	2.8 ± 0.3	3.3 ± 0.1	11.6±1.0

Cellular processing and retention

Cells were seeded in cell-culture dishes with a density of 10^6 cells/dish for all experiments. A set of three dishes was used for each data point.

To study processing of the primary probe, cells were incubated with [^{177}Lu]Lu-$Z_{HER2:342}$-SR-*HP15* (1 nM) for 1 h at 4 °C. The medium was removed, the cells were washed on ice, new medium was added and the cells were placed in a humidified incubator at 37 °C. At 1, 2, 4, 8 and 24 h, the medium was collected, cells were washed and treated with 0.2 M glycine buffer containing 4 M urea, pH 2.0, for 5 min on ice. The acidic solution was collected and cells were additionally washed with 1 mL glycine buffer. The cells were then incubated with 1 mL of 1 M NaOH at 37 °C for 10 min and collected with 1 mL of 1 M NaOH. The radioactivity in acidic fractions was considered as membrane-bound, and in the alkaline fractions as internalized.

For cellular processing and retention of [^{177}Lu]Lu-secondary probes by SKOV3 and BT474, cells were incubated with $Z_{HER2:342}$-SR-*HP15* (1 nM) for 1 h at 4 °C, then the medium was removed, the cells were washed on ice with cold medium, [^{177}Lu]Lu-secondary probe (10 nM in cold medium) was added, and the cells were incubated for 30 min at 4 °C. Then the medium was removed, the cells were washed on ice, new cold medium was added and the cells were placed in a humidified incubator at 37 °C. At 1, 2, 4, 8 and 24 h, a group of three dishes was removed from the incubator and treated as described above.

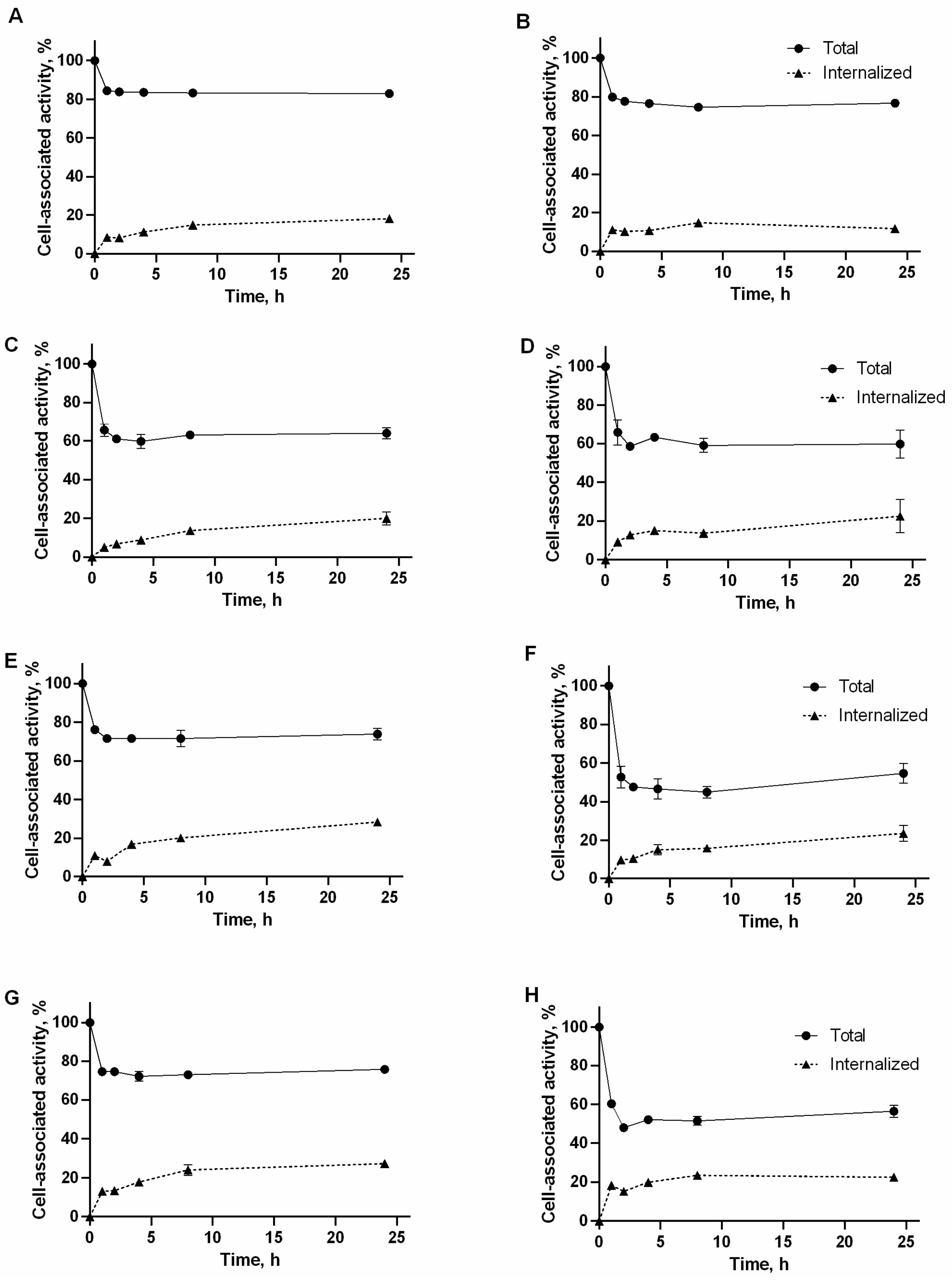

Figure S14. Cellular processing and retention of [^{177}Lu]Lu-ZHER2:342-SR-*HP15* (**A**,**B**) , [^{177}Lu]Lu-*HP16* (**C**,**D**), [^{177}Lu]Lu-*HP17* (**E**,**F**) and [^{177}Lu]Lu-*HP18* (**G**,**H**) by SKOV3 (**A**,**C**,**E**,**G**) and BT474 (**B**,**D**,**F**,**H**) cells after interrupted incubation with labeled compounds. In the case of labeled secondary probes, cells were pre-treated with non-labeled ZHER2:342-SR-*HP15*. Thereafter, cells were incubated with the labeled compound at 4 °C to allow annealing with primary probes. After changing of medium, the cells were incubated at 37 °C. The data are presented as an average value from 3 samples ± SD.

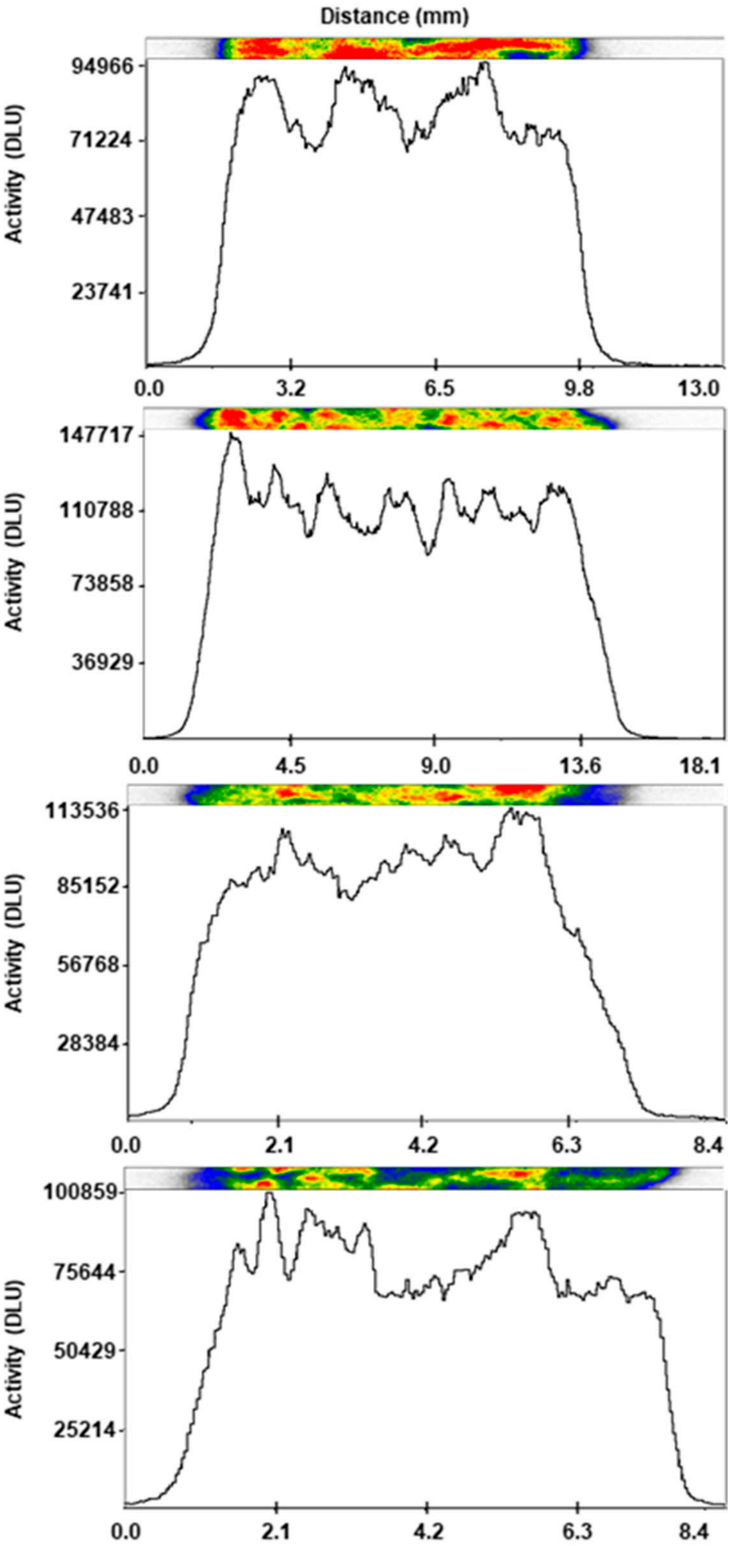

Figure S15. Ex vivo autoradiography of tumor slices of mice bearing HER2-expressing tumors from the pretargeting experiment. Mice were pre-injected with the primary agents, 16 h later they were injected with (A) [^{177}Lu]Lu-*HP16* , (B) [^{177}Lu]Lu-*HP17*, (C) [^{177}Lu]Lu-*HP18*, (D) [^{177}Lu]Lu-*HP2* and were dissected 4 h pi.

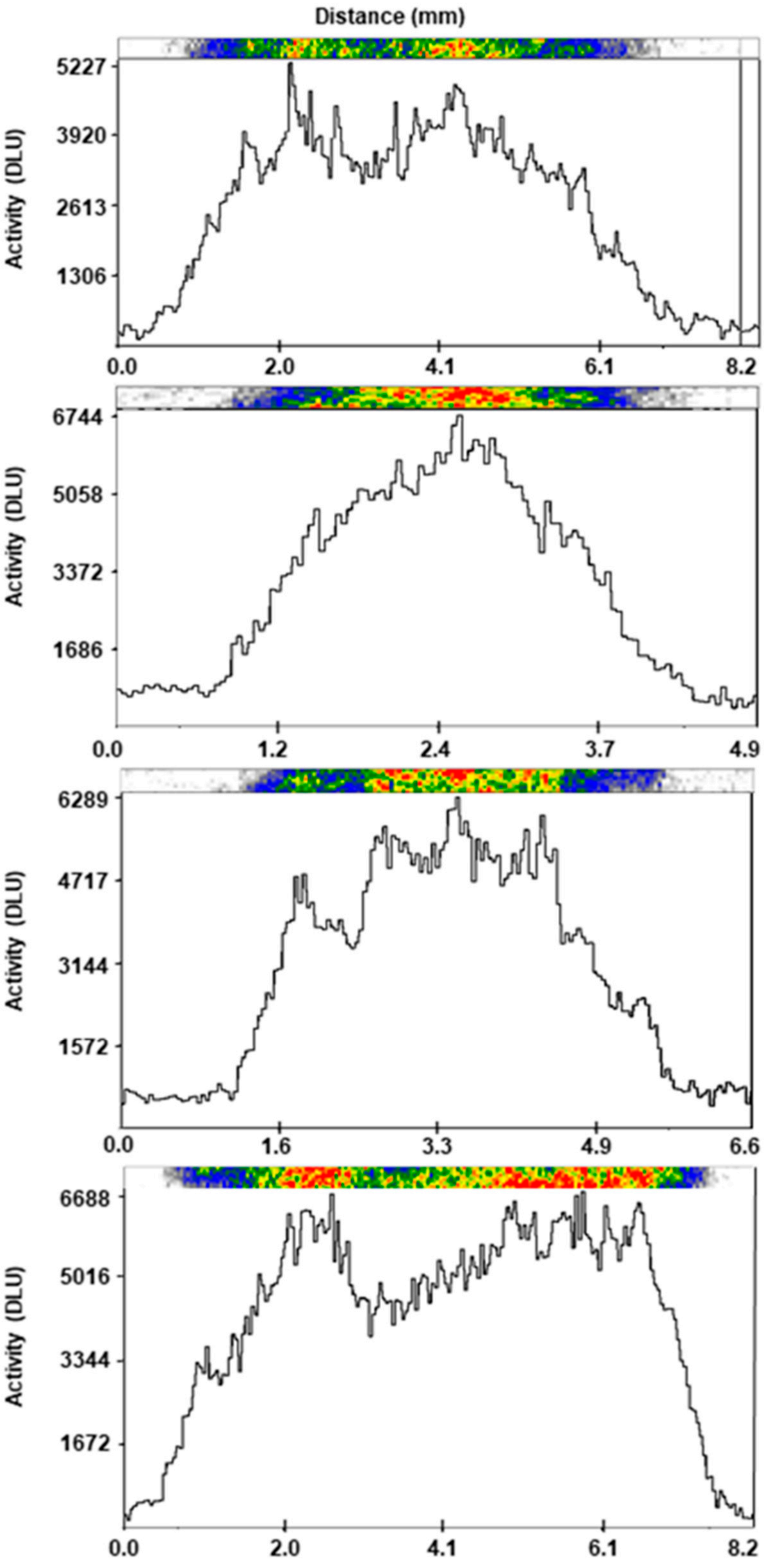

Figure S16. Ex vivo autoradiography of tumor slices of mice bearing HER2-expressing tumors from the pretargeting experiment. Mice were pre-injected with the primary agents, 16 h later they were injected with (**A**) [^{177}Lu]Lu-*HP16*, (**B**) [^{177}Lu]Lu-*HP17*, (**C**) [^{177}Lu]Lu-*HP18*, (**D**) [^{177}Lu]Lu-*HP2* and were dissected 144 h pi.

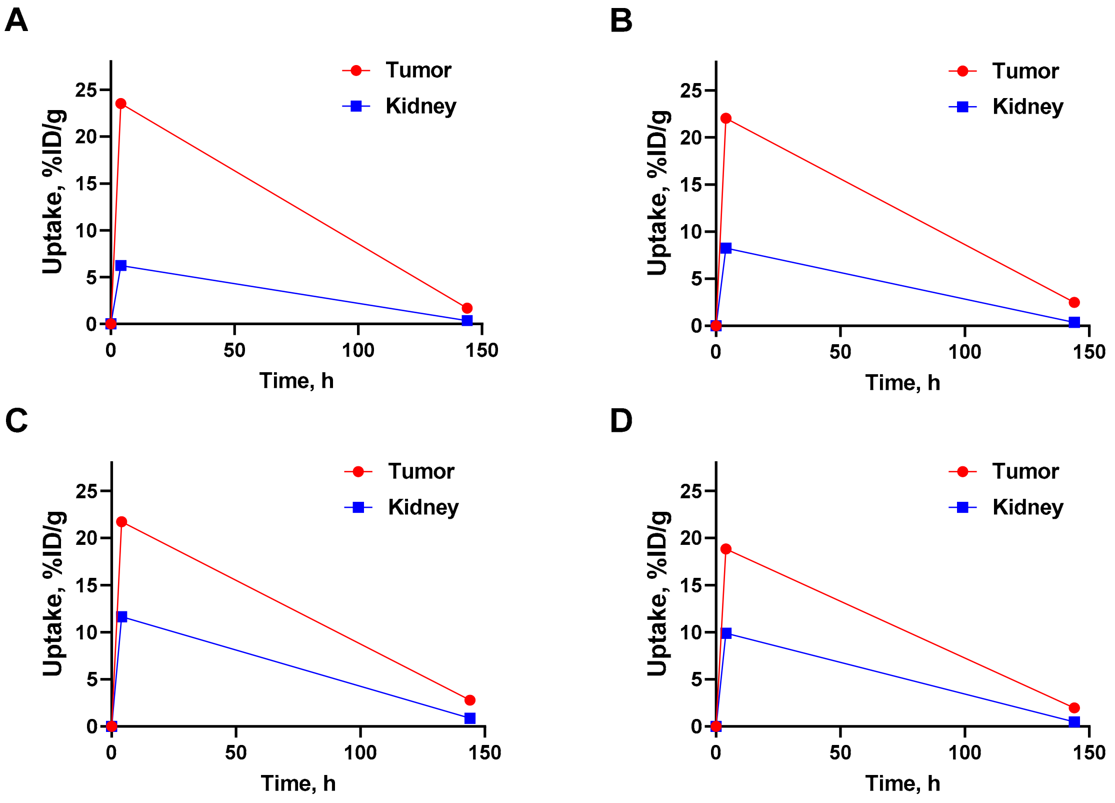

Figure S17. Time-activity plots for (**A**) [177Lu]Lu-*HP16*, (**B**) [177Lu]Lu-*HP17*, (**C**) [177Lu]Lu-*HP18* and (**D**) [177Lu]Lu-*HP2* . Non-decay corrected data for both kidney and tumor were used.

Table S2. Areas under time-activity plot.

	**Area for kid-neys**	**Area for tu-mor**	**Tumor-to-kidney ratio**
[177Lu]Lu-*HP16*	460	1780	3.8
[177Lu]Lu-*HP17*	610	1730	2.8
[177Lu]Lu-*HP18*	890	1730	2.0
[177Lu]Lu-*HP2*	730	1470	2.0
